# Two-color measurements supporting a revised formulation for the refractive index of air

**DOI:** 10.1088/1361-6501/ae6e94

**Published:** 2026

**Authors:** Patrick F Egan

**Affiliations:** National Institute of Standards and Technology, Gaithersburg, MD 20899, United States of America

**Keywords:** refractometry, Ciddor equation, length metrology, thermodynamic metrology

## Abstract

Two-color refractivity measurements are reported for carbon dioxide, oxygen, binary mixtures of nitrogen–oxygen, natural dry air, and water vapor. For the ‘dry’ gases, the measurements were simultaneously performed at 0.6329908μm and 1.542383μm, and cover temperatures $(20<\left.t_{90}<100\right)$ °C and pressures $p<0.5\;{\text{MPa}}^{-1}$. For water vapor, the refractivity ratio was studied for six wavelengths $\left(1.52<\lambda <1.57\right)\mu \text{m}$ relative to a simultaneous measurement made at 0.6329908μm. The carbon dioxide and oxygen measurements establish the molar refractivities and second density virial coefficients more accurately than anything done before. A Sellmeier equation is formulated for oxygen which is about 10^3^ times more accurate than extant knowledge dating from 1932. The nitrogen–oxygen measurements are used to derive the second density cross virial coefficient. The natural dry air measurements provide a targeted refinement to the Ciddor formulation for the refractive index of air, because they establish both a reference value for refractivity and the second density virial coefficient. The water vapor measurements ensure an accurate Ciddor formulation at telecom wavelengths, and guide the implementation of the group index.

## 1. Introduction

Laser wavelength in vacuum λ=c/ν is given by the speed of light in vacuum c divided by frequency ν. In air, the wavelength is reduced λ=c/(nν) by the refractive index n. The majority of applications employing wavelength occur in air. Therefore, knowledge of the refractive index of air is important in many fields. The primary example application is realization of the SI meter via interferometry [1]. Other examples include optical instruments [2], material characterization [3, 4], spectrometry [5, 6], remote sensing [7], and infrasonic metrology [8].

Knowledge of the refractive index of air is usually inferred from measurements of the environmental conditions: pressure p, temperature $t_{90}$, relative humidity ψ, and carbon dioxide content $x_{\text{c}}$. Refractive index may be derived from measurement of these quantities together with a reference formulation $n=f\left(p,t_{90},\psi ,x_{\text{c}}\right)$. In 1996, Ciddor [9] constructed a reference formulation for the refractive index of air, applicable in a wide range of conditions (environmental and laser frequencies). Ciddor’s formulation has stood for the past 30 years, but it has long been suspected that its treatment of water vapor may have shortcomings. Recently, these suspicions have been reified in two unrelated applications of precision interferometry. First, a measurement of water vapor refractivity in the near infrared [10] indicated widely divergent behavior from a $1/\lambda^{2}$ Cauchy equation. (The Ciddor formulation uses a $1/\lambda^{2}$ Cauchy equation for water vapor refractivity.) Second, a geospatial comparison between a dual-wavelength optical distance meter [11] and a global navigation satellite system revealed a signature of water vapor problems in the near infrared.

To remedy these water refractivity problems—and to update several other (minor) aspects—the Ciddor formulation is being revised in a pair of articles. One article will focus on formulation for the optical properties of the constituent gases, which are the primary input to the Ciddor formulation. The other article will focus on the density of air [12], which is a second-order effect in the Ciddor formulation, but has broad application in many fields besides laser wavelength. Both articles rely on new refractometry measurements to fill some gaps in knowledge. These new measurements are reported in this article. Besides their central importance in improving the Ciddor formulation, the measurements reported below are likely to benefit atmospheric science [13], and benchmark thermophysical properties calculated by *ab initio* methods [14].

## 2. Apparatus and analysis

The apparatus is the same as that used by Yang *et al* [15], and comprises a Fabry–Perot (FP) cavity to measure refractivity, a piston gage to generate a calculable pressure, and a capsule-type standard platinum resistance thermometer to measure ITS-90 temperature. The FP cavity is suspended by wires in a pressure vessel. The pressure vessel begins at high vacuum, and the resonance frequency of the cavity is measured. The pressure vessel is filled with a test gas until the piston gage floats and generates a pressure. After a 1500 s wait time for thermal equilibration, the resonance frequency of the cavity is measured once again together with the temperature of the gas. Refractivity is calculated

(1) $$n-1=-\frac{\Delta f}{\nu }+\frac{n\Delta L-2\epsilon_{\psi }}{L},$$

from the fractional change in resonance frequency between vacuum and the generated pressure. The working equation for the refractometer was more formally explained by Yang *et al* [15]. The Δf is the measured change in resonance frequency relative to vacuum, and ν is the vacuum resonance frequency of the cavity. The ΔL/L is the compression in cavity length as the surrounding pressure changes from vacuum to atmosphere. The ΔL/L was deduced via helium measurement [15], and is linear proportional to pressure. For the measurements of water vapor only, equation (1) includes a $\epsilon_{\psi }/L$, which represents the shift in the reflecting surface of a cavity mirror as water vapor adsorbs into the thin-film coating. The $\epsilon_{\psi }/L$ was characterized by Egan and Yang [16], and is nonlinear proportional to relative humidity.

The apparatus has a number of key attributes that enable high accuracy work. The FP cavity is simultaneously interrogated with lasers operating at 0.6329908μm and 1.542383μm, and both measured resonance frequencies are referenced to a frequency comb. The accuracy in the frequency metrology is at the level of 10^−12^, but uncertainty in the distortion error ΔL/L of the cavity limits the accuracy of a refractivity measurement to 2 × 10^−15^ Pa^−1^ [15]. This accuracy corresponds to about 2×10^−10^ uncertainty in the refractive index at ambient pressure, or ${8\times 10}^{-7}(n-1)$ in the refractivity of dry air. The piston gage generating the pressure has had its diameter dimensionally characterized, so that the generated pressure may be calculated [17] via F/A within ${1.9\times 10}^{-6}p$. Finally, the lid of the aluminum pressure vessel protrudes into the FP cavity volume, and has two boreholes: one in the pure gas volume to enclose the cavity mode (the laser beam resonating between the mirrors), and one on the room air side to house the resistance thermometer. This thermal feature ensures that the temperature of the gas interacting with the cavity mode is known within 1 × 10^−6^T [15]. The quadrature sum of these three components—refractivity, pressure, and temperature—represents the ‘baseline uncertainty’ of the apparatus, which is 2.3 × 10^−6^ relative standard uncertainty. A detailed uncertainty budget is given in the supplemental material of Yang *et al* [15]. In this work, the overall measurement uncertainty will combine this baseline uncertainty with the additional contributors specific to each gas, as described in each section below.

Two equations underlie the analyzes described in the sections below. The first is the extended Lorentz–Lorenz equation

(2) $$\frac{n^{2}-1}{n^{2}+2}=A_{\text{R}}\rho \left(1+b_{\text{R}}\rho +c_{\text{R}}\rho^{2}\right),$$

which relates the measured quantity refractive index n to the molar refractivity $A_{\text{R}}$ and the molar density ρ. The refractivity virial coefficients $b_{\text{R}}$ and $c_{\text{R}}$ account for field interactions between the gas molecules. The second equation is the virial equation of state

(3) $$\frac{p}{RT}=\rho \left(1+B_{\rho }\rho +C_{\rho }\rho^{2}\right),$$

which relates the measured quantities pressure p and temperature T to the molar density. The molar gas constant R is a fixed value, and the density virial coefficients $B_{\rho }$ and $C_{\rho }$ account for deviation from ideal gas behavior. By eliminating the common term ρ from the two equations above, the final analysis regresses data to

(4) $$p=\left(n-1\right)𝒜\left[1+(n-1)ℬ+(n-1{)}^{2}𝒞\right]+\epsilon_{p}.$$

The fit coefficient $𝒜=2RT/\left(3A_{\text{R}}\right)$ has dimensions $\text{Pa}\equiv \text{J}\;{\text{m}}^{-3}$, and yields the molar refractivity. The dimensionless fit coefficients ℬ and 𝒞 are lumped products of the density and refractivity virial coefficients that describe real gas behavior. In thermodynamic metrology, ℬ and 𝒞 are sometimes called refractive index gas thermometry (RIGT) virial coefficients [18]. The extra term $\epsilon_{p}$ in equation (4) accounts for offset error in the apparatus, arising from either the piston gage or refractometer. For the present apparatus, typically $\left|\epsilon_{p}\right|\approx (30\pm 50)$ mPa, corresponding to about 3 × 10^−7^ fractional error in either refractivity or pressure near atmospheric conditions. Consequently, the contribution of $\epsilon_{p}$ to the final analysis is very small, but its presence in the regression is physically justified.

The article now proceeds with sections dedicated to each gas studied. Measurement procedure for the four dry gases was identical: carbon dioxide in section 3, oxygen in section 4, the nitrogen–oxygen binary mixtures in section 5, and natural dry air in section 6. All these gases have zero water content and were supplied compressed in cylinders. Consequently, these gases seamlessly integrate with the automated isotherm procedure, mass flow controller, etc [19]. The last gas studied—water vapor—has a low pressure measurement procedure, operating below the $p_{\text{ws}}\approx 2.3\;\text{kPa}$ saturation pressure of water at 20 °C. The water vapor measurements will be described in section 7.

## 3. Carbon dioxide

Carbon dioxide is a minor component of air, with an approximate mole fraction 4 × 10^−4^. Indeed, for refractive index applications less accurate than 10^−7^, the fluctuating carbon dioxide contribution is usually not included in a reference formulation. Nevertheless, carbon dioxide is a strong infrared absorber, and therefore small variations in its concentration have a large effect on the dispersion of air (which is important for many instruments employed in long distance interferometry [20]). Therefore, the motivation to measure carbon dioxide is primarily to establish its molar refractivity $A_{\text{R}}$ at two wavelengths, and to tightly constrain a Sellmeier equation for refractivity.

In addition to the molar refractivity of carbon dioxide, the present work also derives the second density virial coefficient $B_{\rho }$. Improved knowledge about the second density virial coefficient is topical because of the (somewhat) renewed interest in carbon dioxide as a refrigerant [21] and as a working fluid for power generation [22, 23].

### 3.1. Method for CO_2_

Nine carbon dioxide isotherms were acquired $\left(20<t_{90}<100\right)$ °C, with refractivity at two wavelengths simultaneously measured up to p<0.5MPa. The analysis followed the multi-isotherm method outlined by Egan and Yang [19], and data were regressed to equation (4). The molar refractivity was allowed a temperature dependence

(5) $$A_{\text{R}}(T)=A_{303}\left[1+A_{\theta }(T/\text{K}-303)\right].$$

Note that all T in this article refers to the measured ITS-90 value [24]. Additional constraints were placed on the unitless coefficients

(6) $$ℬ=\sum_{i=0}^{3}b_{i}(T/\text{K}-303{)}^{i}\;\text{and}\;𝒞=\sum_{i=0}^{1}c_{i}(T/\text{K}-303{)}^{i}.$$

The choice of polynomial order was guided by *ab initio* calculation of the density virial coefficients [25], which dominate ℬ and 𝒞. For the temperature range of the present work, a cubic should describe ℬ within 0.015%, which is less than half the standard uncertainty of the present measurement.

### 3.2. Molar refractivity of CO_2_

**Figure 1.**
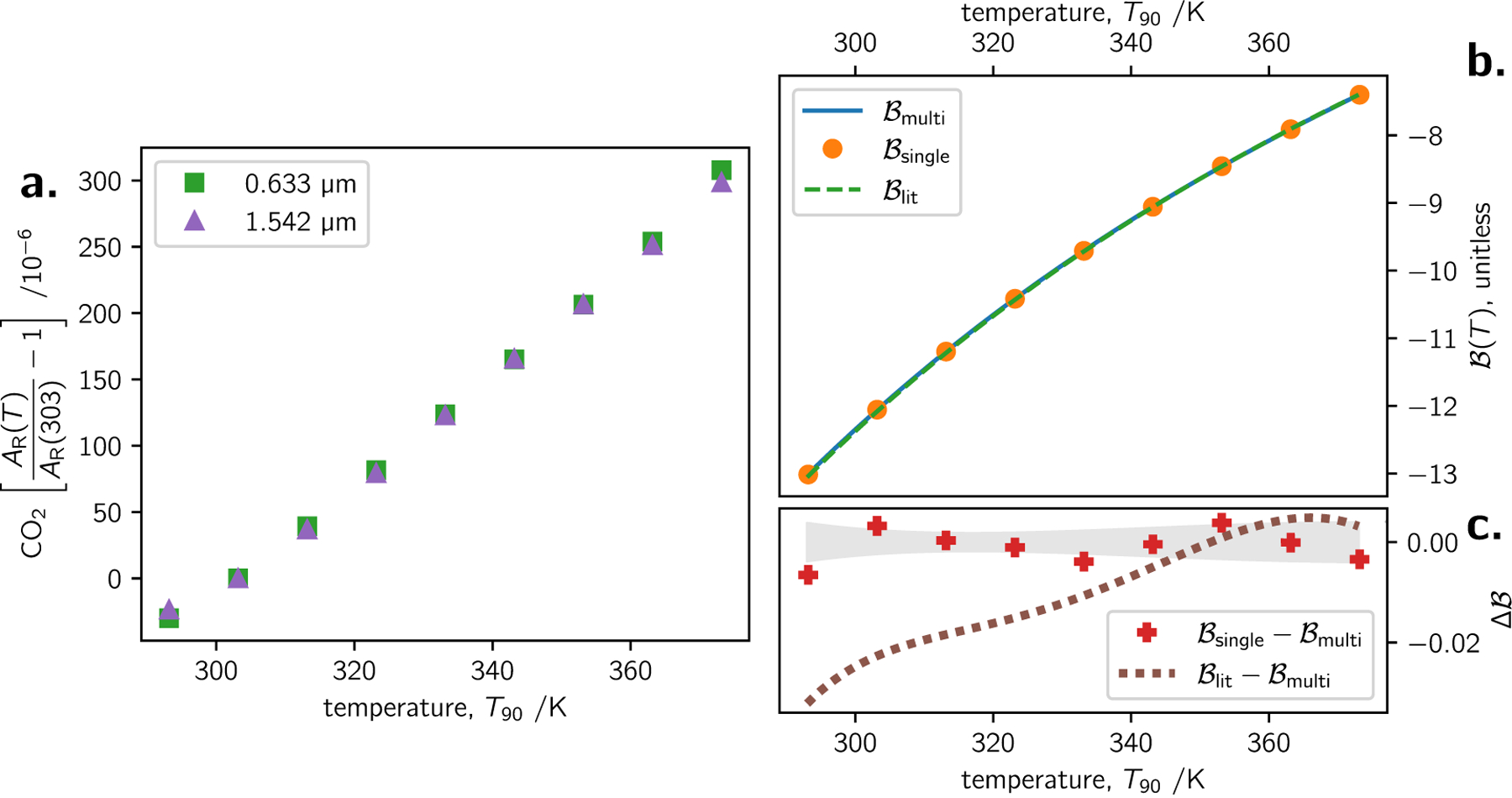
Measurement of carbon dioxide isotherms. (a) Temperature dependence of the molar refractivity. (b) The second RIGT virial coefficient ℬ. (c) Difference between this work and literature for ℬ. The shaded area denotes statistical uncertainty of this work.

For $A_{\text{R}}$ at 0.6329908μm, the analysis identified coefficients for equation (5) as $A_{303}=6.64337(3)\;{\text{cm}}^{3}{\;\text{mol}}^{-1}$ and $A_{\theta }=4.22(9)\times 10^{-6}$. At 1.542383μm, the result was $A_{303}=6.50541(3)\;{\text{cm}}^{3}{\;\text{mol}}^{-1}$ with $A_{\theta }=4.13(9)\times 10^{-6}$. The numbers in parentheses denote statistical uncertainty only; combined measurement uncertainty for the molar refractivity is explained below. The relative change in molar refractivity as a function of temperature for both wavelengths across the nine isotherms is shown in figure 1(a). From the plot, the $A_{\text{R}}(T)$ deviating from linear is perceptible, but the nonlinear trend also has contribution from temperature scale error [26, 27]. However, since the temperature scale error is common to both wavelengths, taking the difference between both $A_{\text{R}}(T)$ clearly shows $A_{\theta }$ is 3.2% larger for the high frequency in this temperature range. The present estimate of $A_{\theta }$ appears the first to date, though there is one inference $A_{\theta }=1.7\times 10^{-6}$ due to Harvey and Lemmon [28], from their reanalysis of Schmidt and Moldover’s [29] capacitor data. Schmidt and Moldover’s original analysis could not detect $A_{\theta }$ in their CO_2_ data; it is reasonable to assume the $A_{\theta }$ deduced by Harvey and Lemmon is limited by the precision of the source data.

For $A_{\text{R}}$, the one measurement of sufficient quality to corroborate the present results is due to Birch [30], who reported $A_{\text{R}}=6.6418(5)\;{\text{cm}}^{3}{\;\text{mol}}^{-1}$ at 0.633μm. Their result is lower than the present work by just over mutual expanded uncertainty.

### 3.3. Second density virial coefficient of CO_2_

Isotherm regression also produces ℬ in equation (4). The ℬ is a single value when regressed on a single isotherm, but follows a model function ℬ(T) when regressed over multiple isotherms. The single- and multi-isotherm results are plotted in figure 1(b), alongside the best knowledge from a literature synthesis [25, 32, 33] denoted ${ℬ}_{\text{lit}}$. Instead of comparing to a literature synthesis, the alternative analysis is to derive [19] the second density virial coefficient

(7) $$B_{\rho }=\frac{3A_{\text{R}}}{2}ℬ+b_{\text{R}}+\frac{A_{\text{R}}}{4},$$

from ℬ and $A_{\text{R}}$ of the present work, plus separate information about the second refractivity virial coefficient $b_{\text{R}}$. The $b_{\text{R}}(T)\equiv \left(b_{\epsilon }+b_{\text{R}}^{\left(2\right)}\sigma^{2}\right)\left(1+b_{\theta }\Delta T\right)$ is composed of the dielectric virial coefficient $b_{\epsilon }$ and its frequency dependence $b_{\text{R}}^{\left(2\right)}$, and this work assumes that both components have the same relative temperature dependence $b_{\theta }$. To compose $b_{\text{R}}$, this work used the measurement due to Achtermann *et al* [32] at 323 K and 0.633μm as the reference value. The temperature and frequency dependencies were constrained by the method outlined in the appendix. So, for carbon dioxide, this work recommends

$$b_{\text{R}}/\left({\text{cm}}^{3}{\;\text{mol}}^{-1}\right)=\left(0.291+0.00575\sigma^{2}\right)\left[1-3.2\times 10^{-3}(T/\text{K}-303)\right].$$

The dimensionless frequency σ=μm/λ has wavelength λ=c/ν given by the speed of light in vacuum divided by frequency. For the dimensions of $b_{\text{R}}$, it is recalled from equation (2) that the often stated second refractivity virial coefficient is $B_{\text{R}}=A_{\text{R}}b_{\text{R}}$.

The derived results for $B_{\rho }(T)$ are tabulated in table 1, where the close agreement with the *ab initio* calculation of Hellmann [25] and the reference equation of state [31] is also evident. Note that the *ab initio* potential surface was adjusted [34] so that the second density virial coefficient derived from it better matched experimental data near ambient temperature. For the temperature range of this work, the *ab initio* calculation without adjustment is about −0.2 cm^3^ mol^−1^ lower than what is listed in table 1. For the adjusted calculation, deviation from measurement does not exceed 0.3%, and the trend in disagreement as a function of temperature is, essentially, the dotted line plotted in figure 1(c). Disagreement is larger when the present work is compared to the unadjusted calculation, but the differences are still well within the mutual expanded uncertainty.

### 3.4. Uncertainty of the CO_2_ results

Throughout this work, the notation u(x) is used to denote the standard uncertainty of the quantity x. Unless otherwise stated, all uncertainties in this work are one standard uncertainty, corresponding to approximately a 68% confidence level.

The molar refractivity $A_{\text{R}}(T)$ expressed by equation (5) has uncertainty contributed from the nominal temperature value plus the temperature dependence. In this work, it is repeated that all temperatures refer to the ITS-90 measured value, which has nonlinear errors of up to 9 mK in this temperature range [26, 27]. Consequently, $A_{\text{R}}(T)$ has uncorrected relative error of up to 2.7 × 10^−5^, which affects both $A_{303}$ and $A_{\theta }$. For $u\left(A_{303}\right)$, the combined uncertainty has three contributors: apparatus baseline (systematic), linear fitting (statistical), and impurity (bias). The apparatus baseline uncertainty is $2.3\times 10^{-6}A_{\text{R}}$ and the fit uncertainty is $3.6\times 10^{-6}A_{\text{R}}$. Fit uncertainty is larger than the baseline uncertainty due to the temperature scale error plus the fact that a linear trend is inadequate to describe $A_{\text{R}}(T)$. The carbon dioxide cylinder was specified as 99.9995% purity, with the main contaminant nitrogen stated not to exceed 5 × 10^−6^. The presence of nitrogen at this level may bias the carbon dioxide measurement low by $1.6\times 10^{-6}A_{303}$. So, summing the three contributors, the combined standard uncertainty $u\left(A_{303}\right)$ is $4.6\times 10^{-6}A_{303}$. The $u\left(A_{\theta }\right)$ is 8.0% with the contribution from the temperature scale error an order of magnitude larger than fit statistics. (Data supporting this article are available [35], and may be reanalyzed to correct the temperature scale error [26, 27].)

Uncertainty in the derived $B_{\rho }$ has two main components: statistical variability in ℬ from the multi-isotherm regression plus systematic conversion error from $b_{\text{R}}$. The u(ℬ), plotted as the shaded area of figure 1(c), was estimated by Monte Carlo methods as outlined by Egan and Yang [19]. From equation (7), the equivalent statistical $u\left(B_{\rho }\right)$ is the shaded area multiplied by $3A_{\text{R}}/2\approx 9.9{\;\text{cm}}^{3}{\;\text{mol}}^{-1}$. The contribution from $u\left(b_{\text{R}}\right)$ assumes 10% error in $b_{\text{R}}$, and is based on a table entry from Achtermann *et al* [32] which does not provide an explanation for the uncertainty. The magnitude of the statistical and systematic components are similar throughout the temperature range. The combined uncertainty given in table 1 adds in quadrature the Monte Carlo component and the systematic conversion error.

## 4. Oxygen

Knowledge about the molar refractivity $A_{\text{R}}$ of oxygen is scarce, and it impacts the Ciddor revision in several indirect ways. First, in principle, the refractive index of air may be calculated from the sum of its constituents at specific mole fractions. Comparing this sum of constituents to a measurement of natural air offers an important consistency check. Until now, imprecision in the $A_{\text{R}}$ of oxygen has prevented this sum of constituents calculation at wavelengths distant from 0.633μm.

A second interest in $A_{\text{R}}$ is that, for the nitrogen–oxygen binary mixtures measured in section 5, knowledge of the composition is essential (e.g. to derive the second density cross-virial coefficient). A refractometer may function as a binary gas analyzer if the $A_{\text{R}}$ of each constituent is precisely known.

Finally, from the multi-isotherm analysis of oxygen, the second density virial coefficient may be derived. Information about this property has become important for accurate flow metering [36] and process control in semiconductor manufacturing.

### 4.1. Method for O_2_

The oxygen measurements were performed similar to carbon dioxide. Nine isotherms were acquired $\left(20<t_{90}<100\right)$ °C, with refractivity at two wavelengths simultaneously measured up to p<0.5MPa. The analysis followed equation (4), and allowed for a temperature dependence for the molar refractivity via equation (5). Again, *ab initio* calculations for oxygen [37] guided the same order polynomials for ℬ and 𝒞 in equation (6), and indicated that a cubic should describe ℬ within 0.015%.

**Figure 2.**
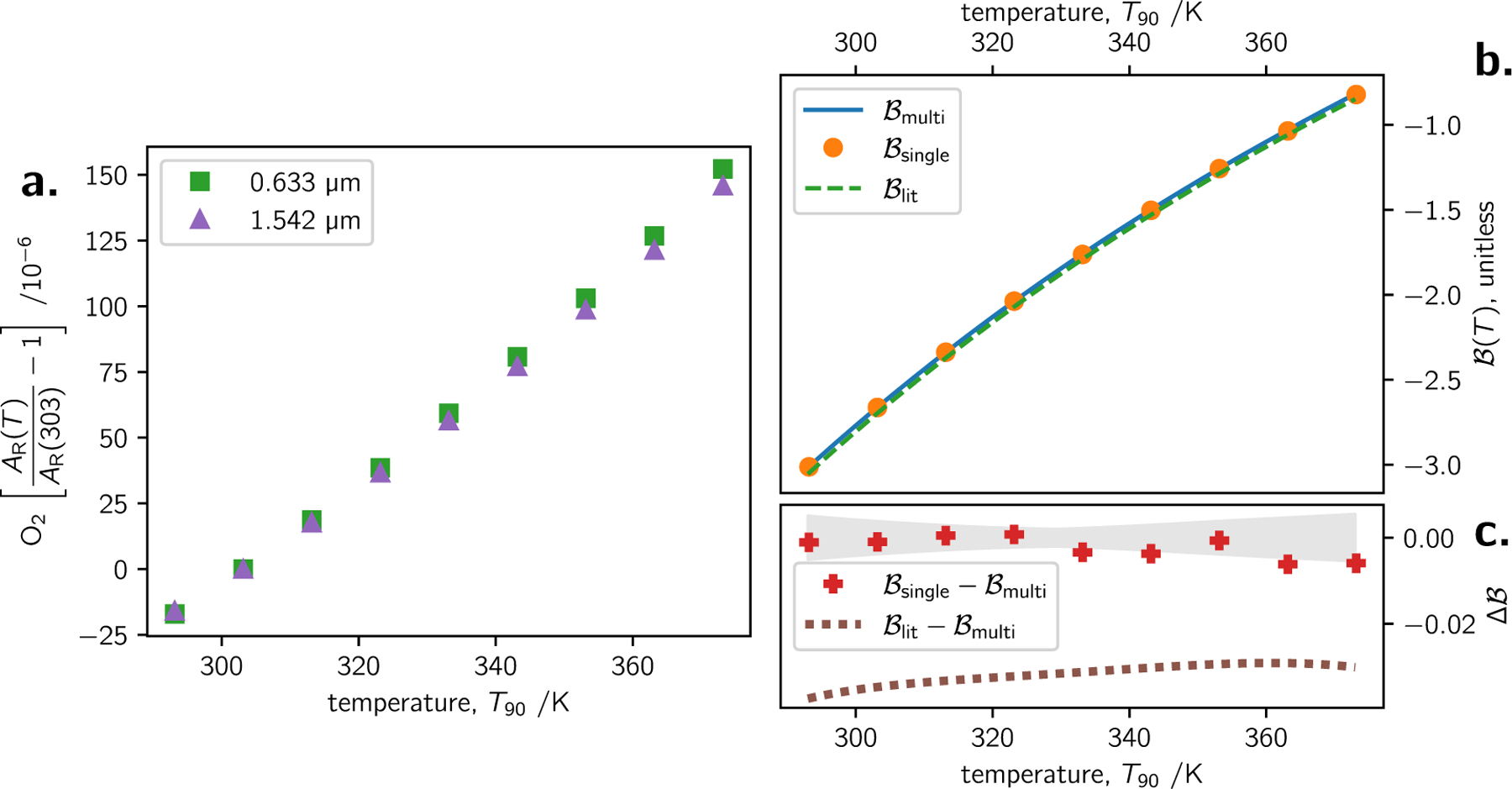
Measurement of oxygen isotherms. (a) Temperature dependence of the molar refractivity. (b) The second RIGT virial coefficient ℬ. (c) Difference between this work and literature for ℬ. The shaded area denotes statistical uncertainty of this work.

The summary results of the oxygen isotherms are shown in figure 2, and the presentation is identical to what has been explained in section 3 for carbon dioxide.

### 4.2. Molar refractivity of O_2_

For $A_{\text{R}}$ in equation (5), the analysis identified $A_{303}=4.027716(7)\;{\text{cm}}^{3}{\;\text{mol}}^{-1}$ and $A_{\theta }=2.11(4)\times 10^{-6}$ at 0.633μm. At 1.542μm, the result was $A_{303}=3.968990(7)\;{\text{cm}}^{3}{\;\text{mol}}^{-1}$ with $A_{\theta }=2.02(5)\times 10^{-6}$. The numbers in parentheses denote statistical uncertainty only, and the combined measurement uncertainty is given below.

The $A_{303}$ results will be placed in the literature context of the subsection below which constructs a Sellmeier equation. Here, the temperature dependency $A_{\theta }$ may be compared to several sources. Hohm and Kerl [38] found $A_{\theta }=2.2\times 10^{-6}$ for eight optical measurements at 0.633μm carried out (300<T<900) K. Hohm and Kerl do not provide an uncertainty statement, and their high temperature data is inconsistent with theory [39]. However, the root-mean square deviation from their fit was $3\times 10^{-4}A_{303}$, which suggests 25% uncertainty on $A_{\theta }$. May *et al* [40] derived $A_{\theta }=2.4(9)\times 10^{-6}$ from three isotherms measured at microwave frequency, which is consistent with the present work. Buldakov *et al* [39] estimated $A_{\theta }=2.0\times 10^{-6}$ near room temperature and suggest that their calculation may be as accurate as 3.6%. Sharipov *et al* [41] calculated $A_{\theta }=2.2\times 10^{-6}$ near room temperature. An excellent agreement between calculation and measurements from the present apparatus for nitrogen [19] has been noted.

### 4.3. Second density virial coefficient of O_2_

To derive [19] $B_{\rho }$ requires separate information about the second refractivity virial coefficient $b_{\text{R}}$. For oxygen, information on $b_{\text{R}}$ is scarce, and does not appear to be more advanced than the heuristic estimate of Hohm [33]. For the frequency dependence, the two-color measurement in this work indicates Hohm’s $b_{\text{R}}^{\left(2\right)}$ is 50% too small. Hohm does not state the temperature dependence, but qualitatively groups oxygen with several other species exhibiting $b_{\text{R}}(T)$ negative proportional to temperature. However, the present work indicates a positive slope. Hohm’s incorrect sign on $b_{\text{R}}(T)$ might originate in their spurious choice for $A_{\text{R}}(T)$. Their choice was based on imprecise data [38], which is inconsistent with rigorous theory at high temperatures [39], and their quadratic fit used for $A_{\text{R}}(T)$ has an inflection at 136 K. In any case, this work uses

$$b_{\text{R}}/\left({\text{cm}}^{3}{\;\text{mol}}^{-1}\right)=\left(0.194+0.00641\sigma^{2}\right)\left[1+1.4\times 10^{-3}(T/\text{K}-303)\right]$$

with an assumption of 50% uncertainty. The recommendation is tied to Hohm’s $b_{\epsilon }$ estimate, but the $\sigma^{2}$ and T dependencies are derived from this work; see the appendix.

The derived result for $B_{\rho }$ is tabulated in table 2. Despite the obvious weakness in knowledge of $b_{\text{R}}$, there is close agreement with the *ab initio* calculation of Hellmann [25] and the reference equation of state [42]. Deviation between measurement and calculation does not exceed 0.25 cm^3^ mol^−1^, and is well within mutual uncertainty.

### 4.4. Uncertainty of the O_2_ results

The molar refractivity of oxygen measured at two wavelengths is described by equation (5). For $u\left(A_{303}\right)$, contributions for fit statistics and impurity of the oxygen are added to the $2.3\times 10^{-6}A_{\text{R}}$ apparatus baseline uncertainty. Uncertainty from fit statistics is large $\left(1.8\times 10^{-6}A_{R}\right)$ because the data is analyzed on ITS-90. The oxygen cylinder was specified as 99.999% purity, with concentrations of nitrogen, argon, and krypton not to exceed 5 × 10^−6^. Impurities from nitrogen and argon have little effect, because of their similar $A_{\text{R}}$ to oxygen. However, krypton is problematic, and could bias the oxygen measurement high by $4.0\times 10^{-6}A_{R}$. The combined standard uncertainty for the oxygen result is $7.2\times 10^{-6}A_{303}$.

Measurement uncertainty in $A_{\theta }$ is dominated by the temperature scale error, which affects both statistical and systematic uncertainties. Analyzing $A_{\theta }$ on ITS-90 has 9% and 2.4% respective systematic and statistical uncertainties, giving a combined standard uncertainty $9.4\times 10^{-2}A_{\theta }$. Here, systematic uncertainty refers to how much the $A_{\theta }$ changes when analyzed with the $T-T_{90}$ correction [26] applied.

For the derived $B_{\rho }$, the statistical contribution was estimated by Monte Carlo methods as outlined by Egan and Yang [19], and is plotted as the shaded area of figure 2(c). The systematic conversion error assumes 50% uncertainty in $b_{\text{R}}$, and is based on how much the Hohm estimate [33] of the frequency dependence $b_{\text{R}}^{\left(2\right)}$ differs from what is observed in this work. Obviously, assuming uncertainty in the absolute value $u\left(b_{\text{R}}\right)$ based what is observed about a second-order influence is risky, and imperfect knowledge about $b_{\text{R}}$ is the weakest aspect for the $B_{\rho }$ result. However, the assumption made in this work has quasi-support from an inference of $b_{\epsilon }$ by the microwave measurements of May *et al* [40]; their fit value is only 18% smaller than the assumption of the work. Unfortunately, the May *et al* estimate of $b_{\epsilon }$ depends on $B_{\rho }$, and their choice of $B_{\rho }$ significantly disagrees with modern estimates (e.g. [37]). Moreover, the May *et al* apparatus was relatively imprecise; for second-order dielectric properties derived from their oxygen data, they cautioned that ‘the values of the parameters $b_{\epsilon }\ldots$ have little physical significance.’ In any case, the combined uncertainty given in table 2 adds in quadrature the Monte Carlo component and the systematic conversion error. The systematic component is an order of magnitude larger than the statistical error, which explains why the combined uncertainty is so large (i.e. compared to carbon dioxide).

### 4.5. Sellmeier equation for $n_{\text{os}}-1$

Current knowledge about oxygen dispersion appears to date from 1932 [43]. Given the importance of oxygen to atmospheric science [13], it is worthwhile to formulate the present two-color result into a Sellmeier equation

(8) $$10^{8}\left(n_{\text{os}}-1\right)=\frac{5627886.8}{281.0-\sigma^{2}}+\frac{197258.6}{45.4-\sigma^{2}},$$

at standard conditions 20 °C and 100 kPa, and recall σ=μm/λ is a dimensionless frequency. (The subscript notation $n_{\text{os}}$ is after Ciddor [9], with ‘o’ denoting oxygen and ‘s’ standard conditions.)

**Figure 3.**
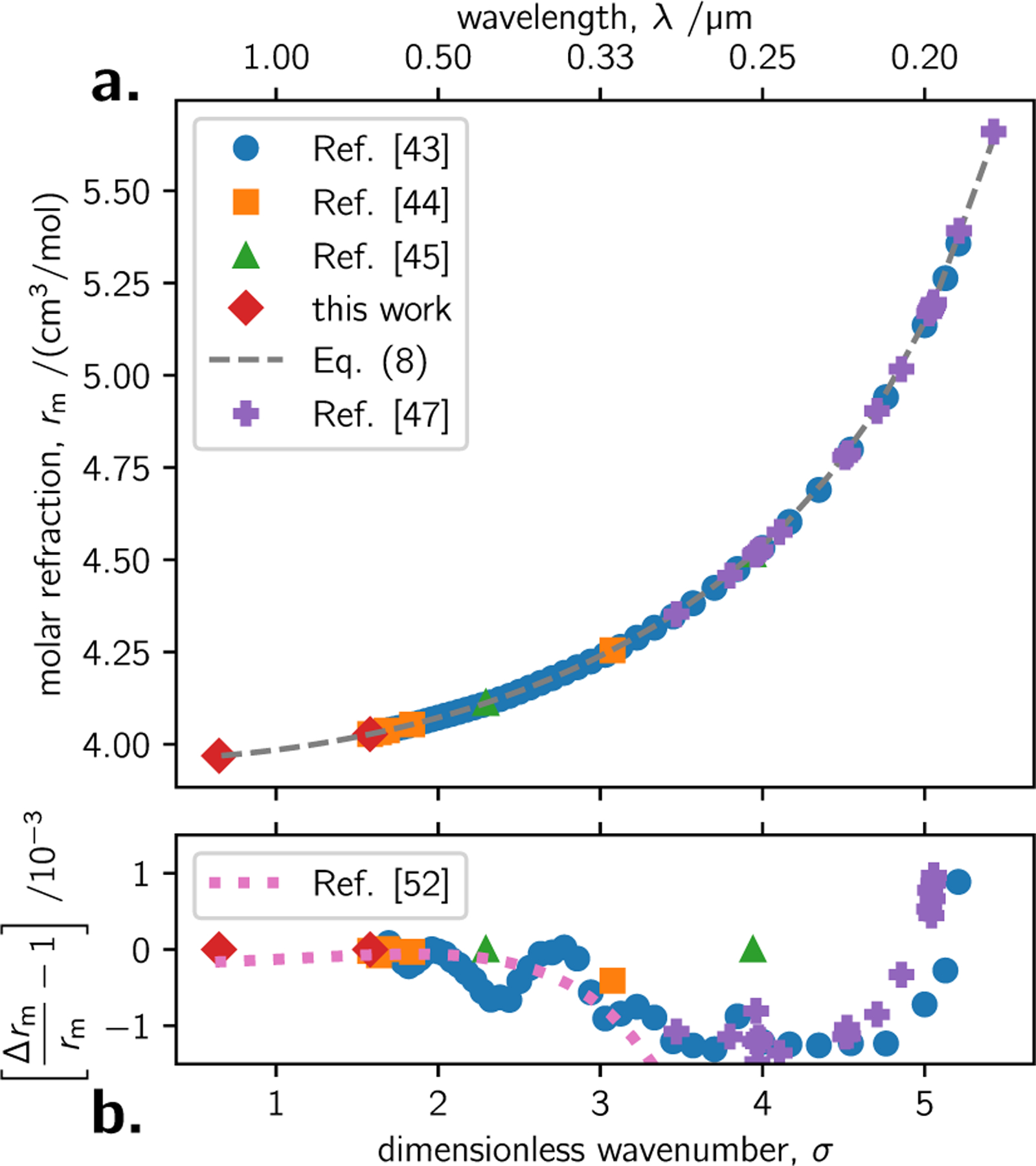
Dispersion of oxygen. (a) Results from this work and literature data constrain the Sellmeier equation (8). (b) Fractional residuals of the measurement data from equation (8). The macroscopic quantity molar refraction $r_{\text{m}}$ is defined in equation (13).

The Sellmeier equation is shown in figure 3(a) together with the data selection upon which it is based. The selection includes:

1. The canonical visible and ultraviolet multiwavelength measurements due to Ladenburg and Wolfsohn [43]. A $10^{-3}(n-1)$ uncertainty was assigned. The Ladenburg and Wolfsohn data intersect the present work at 0.633μm within this uncertainty, and no normalization was applied.
2. Five visible wavelength measurements due to Hohm [44]. The parenthesized notation of Hohm suggested a $10^{-4}(n-1)$ uncertainty assignment. A $-2.1\times 10^{-3}(n-1)$ offset was added to the data to make it intersect the present work at 0.633μm. (A previous measurement by the same group [38] would have required an offset correction an order of magnitude smaller.)
3. The two-color measurement of this work at 1.542μm and 0.633μm. The uncertainty of this work is $7.2\times 10^{-6}(n-1)$.
4. A refractivity ratio between 0.436μm and 0.254μm due to Keeling *et al* [45]. The ratio was incorporated iteratively, first evaluating a provisional equation (8) at 0.436μm, then using the ratio 1.097922 to project a new refractivity at 0.254μm, and finally refitting equation (8). A $10^{-5}(n-1)$ uncertainty was assigned to the projected refractivity.

The fit was weighted by the reciprocal of uncertainty. Equation (8) is consistent within $10^{-6}(n-1)$ of the two-color measurement of this work and the Keeling *et al* ratio. Consistency at this level is expected, given the precision of both apparatuses. This level of consistency is 10^3^ better than the ordinate scale in figure 3(b), which shows fractional residuals between selected data and equation (8). However, the imprecision of the Ladenburg and Wolfsohn data means that the fit is not robust. For example, it is also possible to fit the data selection with a Cauchy equation based on the dipole oscillator strength distribution [46]. Describing the selected oxygen data in the ultraviolet evidently requires three free parameters ($\sigma^{0},\sigma^{2}$, and $\sigma^{4}$) when the remaining coefficients up to $\sigma^{12}$ are set as fixed terms. Consistency of $2.5\times 10^{-6}(n-1)$ between this work and Keeling *et al* is attained, but the zero-frequency extrapolation differs by $7.7\times 10^{-6}(n-1)$ from what the Sellmeier equation indicated. It is unclear if this disagreement arises from nonlinearity in the Ladenburg and Wolfsohn data (which would be problematic for a Cauchy equation), or from inaccuracy of the fixed Cauchy coefficients of the dipole oscillator strength distribution. In any case, for the present data situation, the Sellmeier equation has the more robust fit properties. [Also plotted in figure 3 are ultraviolet data due to Smith *et al* [47], which shows a $10^{-3}(n-1)$ nonlinearity similar to that of Ladenburg and Wolfsohn. The Smith *et al* data were not included for the Sellmeier fit.]

In addition to the lack of fit robustness, equation (8) may not correctly describe the low frequency behavior because oxygen has significant microwave absorption [48] near 60 GHz (or $\sigma =2\times 10^{-4}$). This feature is relevant because it precludes a direct comparison of the present results with the high accuracy microwave work of May *et al* [40]. By transforming [49] the HITRAN absorption line lists [50] into refractive index, it can be shown that the absorption causes microwave refractivity to depart from equation (8) by $2.7\times 10^{-3}(n-1)$ at the $\sigma =3\times 10^{-5}$ operating frequency of May *et al*. Effectively, an optical measurement accesses the electric polarization, whereas a microwave measurement probes the sum of electric and orientational polarization, with the latter arising from photons coupling into the magnetic dipole moment. The molar polarization at microwave frequency should be $2.7\times 10^{-3}r_{\text{m}}$ larger than an optical measurement extrapolated to zero frequency. The result of May *et al* is $3.6\times 10^{-3}(n-1)$ higher than equation (8). This offset between a microwave measurement and the Sellmeier equation based on optical measurements is nominally consistent with the predicted [49] influence of absorption.

By contrast, equation (8) should be accurate throughout the optical frequency range. The weak (resolvable) oxygen absorption near 0.76μm perturbs [49] the *phase* refractive index at the level of $1\times 10^{-5}(n-1)$, but the effect is localized. Therefore, comparisons of equation (8) with other optical results now described should be unambiguous. Birch [30] measured $A_{\text{R}}=4.0278(3)\;{\text{cm}}^{3}{\;\text{mol}}^{-1}$ with a refractometer operating at 0.633μm. The agreement between this work and Birch is excellent, and increases confidence in the present result. Another measurement by Zhang *et al* [51] at 0.800μm is lower than equation (8) by $4.5\times 10^{-4}(n-1)$, or more than five times the mutual expanded uncertainty. The inconsistency of their oxygen result with what was known about air has been discussed [52]. The Zhang *et al* results for carbon dioxide are also discrepant with the Sellmeier equation produced from this work, and problems with their nitrogen and argon results have been pointed out before [10].

Finally, plotted in figure 3(b) is the difference between equation (8) and an inferred Sellmeier equation (single oscillator) due to Křen [52]. Křen inferred a Sellmeier equation for oxygen based on what was known about the dispersion of air (plus the necessary adjustments for nitrogen, argon, and carbon dioxide). At 0.633μm, Křen’s agreement with equation (8) is within $8\times 10^{-5}(n-1)$, but at 1.542μm disagreement increases to $1.7\times 10^{-4}(n-1)$. Evidently, the inferred Sellmeier equation is not metrology-grade. (That is, at ambient conditions, the corresponding error in refractive index is almost 5 × 10^−8^ in the near-infrared; error is significantly larger as λ<0.4μm).

## 5. Nitrogen–oxygen binary mixtures

In the composition of dry air, the sum of nitrogen and oxygen accounts for over 99% of the mole fraction. In planning the scope of work to revise Ciddor, it was believed that measuring a N_2_ + O_2_ binary mixture close in composition to dry air would provide the most convenient and wide-ranging information about dry air. The conceptual idea was to measure the molar refractivity and the second density virial coefficient of the binary mixture. Then, by post-analysis, we infer equivalent values by adjusting the measured results to the mole fraction of dry air.

As the work progressed, it was discovered that natural dry air in compressed cylinders is available as a research item. The initial conceptual idea was abandoned, and data on the N_2_ + O_2_ binary mixture became less than essential to the Ciddor revision. Nevertheless, binary mixture results are of interest to other fields (i.e. calibration fluids for densimeters [53], mixture modeling in equations of state), and the measurements are reported here for this reason.

### 5.1. Method for N_2_ + O_2_

Two cylinders of nitrogen–oxygen binary mixtures were procured, and were specified to have mole fractions of water vapor and carbon dioxide less than 2 × 10^−6^, as were total hydrocarbons. For the N_2_ : O_2_ composition, the mixtures targeted 0.79 : 0.21 and 0.21 : 0.79. (The former blend is sometimes traded as ‘zero grade air’ or ‘synthetic air.’ Gas producers typically specify 2% tolerance on the mole fractions for this type of blend.)

A refractometer may function as a binary gas analyzer. The molar refractivity of a mixture

(9) $$A_{\text{mix}}=\sum_{i}A_{i}x_{i},$$

is the sum of mole fraction $x_{i}$ times molar refractivity $A_{i}$ for each constituent. For a binary mixture, it follows

(10) $$x_{\text{n}}=\frac{A_{\text{mix}}-A_{\text{o}}}{A_{\text{n}}-A_{\text{o}}}.$$

The $A_{\text{mix}}$ is the measured quantity. The reference properties for the constituents have been established for nitrogen $A_{\text{n}}$ by Yang *et al* [15], and for oxygen $A_{\text{o}}$ in section 4. By refractometry, the nitrogen mole fraction of the binary mixtures was found to be: $x_{\text{n}}=0.78706(2)$ and $x_{\text{n}}=0.19971(2)$. For the remainder of this section, each mixture will be identified by its nominal nitrogen mole fraction $x_{\text{n}}$. (Note that $A_{\text{mix}}$ refers to the zero density limit; i.e. $x_{\text{n}}$ has been estimated after five days’ measuring an isotherm and regressing data to zero pressure. The $x_{\text{n}}$ may be deduced instantaneously from a measurement of n-1, but the governing equation would need to account for partial densities and interaction effects, and would be more complicated than above.)

Similar to carbon dioxide and oxygen above, nine isotherms were acquired ($20<t_{90}<100$) °C, with refractivity at two wavelengths simultaneously measured up to p<0.5MPa. Isotherms were analyzed using the same multi-isotherm regression, as described by equation (4) through equation (6).

### 5.2. Molar refractivity of N_2_ + O_2_

For the $x_{\text{n}}\approx 0.787$ binary mixture, the multi-isotherm analysis found the equation (5) coefficients to be $A_{303}=4.35707(3)\;{\text{cm}}^{3}{\;\text{mol}}^{-1}$ and $A_{\theta }=1.4(2)\times 10^{-6}$ at 0.633μm. At 1.542μm, the result was $A_{303}=4.30563(3)\;{\text{cm}}^{3}{\;\text{mol}}^{-1}$ with $A_{\theta }=1.3(2)\times 10^{-6}$. For the $x_{\text{n}}\approx 0.200$ binary mixture, the results were $A_{303}=4.11136(3)\;{\text{cm}}^{3}{\;\text{mol}}^{-1}$ and $A_{\theta }=1.9(2)\times 10^{-6}$ at 0.633μm. At 1.542μm the result was $A_{303}=4.05448(3)\;{\text{cm}}^{3}{\;\text{mol}}^{-1}$ with $A_{\theta }=1.8(2)\times 10^{-6}$. The numbers in parentheses denote statistical uncertainty only, and the combined measurement uncertainty is given below.

### 5.3. Second density virial coefficient for N_2_ + O_2_

As with the other pure gases studied in previous sections, deriving the second density virial coefficient of the nitrogen–oxygen mixture must assume something about $b_{\text{R}}$ in equation (7). For a binary mixture, the second refractivity virial coefficient

(11) $$b_{\text{mix}}=x_{\text{n}}^{2}b_{\text{nn}}+2x_{\text{n}}x_{\text{o}}b_{\text{no}}+x_{\text{o}}^{2}b_{\text{oo}},$$

has combined effects of the pure substance virial coefficients $b_{\text{nn}}$ and $b_{\text{oo}}$, plus a ‘cross virial’ $b_{\text{no}}$. For a nitrogen-oxygen mixture, nothing appears known about the refractivity cross virial $b_{\text{no}}$. Lacking other information, the reasonable assumption is $b_{\text{no}}\approx \left(b_{\text{nn}}+b_{\text{oo}}\right)/2$, and so equation (11) becomes $b_{\text{mix}}\approx x_{\text{n}}b_{\text{nn}}+x_{\text{o}}b_{\text{oo}}$. (This arithmetic mean assumption contributes to $u\left(B_{\text{mix}}\right)$, and will be returned to in the uncertainty section below.) The oxygen term $b_{\text{oo}}$ has been stated in section 4, and is largely based on Hohm [44] with an assigned 50% uncertainty. The nitrogen term would be

$$b_{\text{nn}}/\left({\text{cm}}^{3}{\;\text{mol}}^{-1}\right)=\left(0.195+0.00211\sigma^{2}\right)\left[1+0.0\times 10^{-3}(T/\text{K}-303)\right],$$

which is based on the Achtermann *et al* [32] measurements at 0.633μm which claimed 7% uncertainty. The $\sigma^{2}$ and T dependencies were optimized by the method described in the appendix. For nitrogen, a $b_{\text{R}}(T)$ trend is not detectable within the precision of the method.

**Figure 4.**
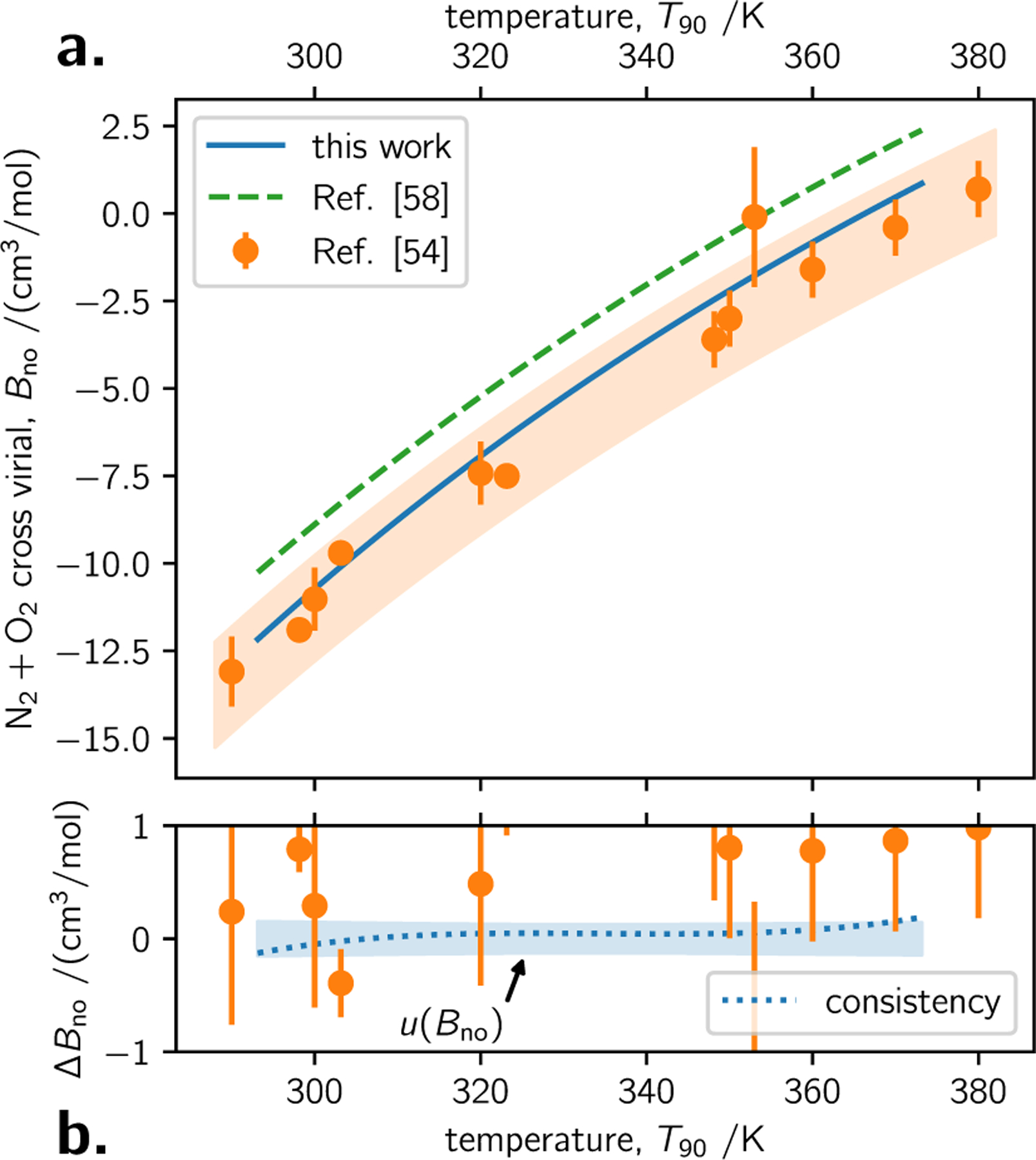
Second density cross virial coefficient $B_{\text{no}}$ of N_2_ + O_2_. Markers with errorbars are taken from the data compilation [54], and the shaded area in (a) shows uncertainty of the suggested fit function. For the residuals plot in (b), the shaded area denotes standard uncertainty of this work. The dotted line ‘consistency’ shows the difference in $B_{\text{no}}$ inferred from the two binary mixtures $x_{\text{n}}\approx 0.787$ and $x_{\text{n}}\approx 0.200$.

The results for $B_{\text{mix}}$ derived from two blends of nitrogen-oxygen are listed in table 3. Perhaps most interesting is to derive the second density cross virial coefficient $B_{\text{no}}$ from results of the two mixtures. Similar to equation (11), the second density virial coefficient of a binary mixture $B_{\text{mix}}=x_{\text{n}}^{2}B_{\text{nn}}+2x_{\text{n}}x_{\text{o}}B_{\text{no}}+x_{\text{o}}^{2}B_{\text{oo}}$ has pure component contributions plus a cross virial. The pure component virial coefficient for nitrogen $B_{\text{nn}}$ is accurately known from Egan and Yang [19], and the oxygen coefficient $B_{\text{oo}}$ was established in section 4. Consequently, the cross virial

(12) $$B_{\text{no}}=\frac{B_{\text{mix}}-\left[x_{\text{n}}^{2}B_{\text{nn}}+{\left(1-x_{\text{n}}\right)}^{2}B_{\text{oo}}\right]}{2x_{\text{n}}\left(1-x_{\text{n}}\right)},$$

may be derived from the $B_{\text{mix}}$ data of table 3. The result is described by the cubic

$$B_{\text{no}}/\left({\text{cm}}^{3}{\;\text{mol}}^{-1}\right)=-10.062+0.19300(T/\text{K}-303)-5.388\times 10^{-4}(T/\text{K}-303{)}^{2}-3.155\times 10^{-7}(T/\text{K}-303{)}^{3},$$

and is plotted in figure 4(a). The standard uncertainty on $B_{\text{no}}$ is 0.13 cm^3^ mol^−1^ on average, and is described in the next subsection. Numerical values for equation (12) evaluated at individual temperatures are given on the rightmost side of table 3.

In figure 4(a), the present work is compared with a data compilation by Dymond *et al* [54], which is largely based on inferred values due to Hall and Iglesias-Silva [55], plus three values from Martin *et al* [56] and one from Fostiropoulos *et al* [57]. (The $B_{\text{no}}$ from Hall and Iglesias-Silva are not based on measurements of an N_2_ + O_2_ binary mixture; rather, $B_{\text{no}}$ was extracted from what was known about air in 1954, together with a multitude of adjustments to remove the influence of argon and carbon dioxide.) Agreement between the present work and older data is good, and errorbars generally overlap with the present work. Dymond *et al* [54] offer a model function with a standard uncertainty of 1.5 cm^3^ mol^−1^, and this function is depicted by the shaded area figure 4(a). Also plotted in figure 4(a) is the cross virial coefficient derived from an *ab initio* potential energy surface due to Bartolomei *et al* [58]. The plotted line is a cubic fit to their tabulated data for (240<T<403) K. In the range (293<T<373) K, Bartolomei *et al* differs from the present work by $\Delta B_{\text{no}}\approx [-1.86+0.0062(T/\text{K}-303)]{\;\text{cm}}^{3}{\;\text{mol}}^{-1}$. Bartolomei *et al* recommended that their calculation be taken as reference data for T>500K; adding a correction factor based on this work might improve their recommendation.

The weakest point of the present work is $B_{\text{oo}}$ in equation (12); the weakness is caused by scarce information on oxygen’s second refractivity virial coefficient. However, for the $x_{\text{n}}\approx 0.787$ mixture, replacing $B_{\text{oo}}$ with the *ab initio* value [37] only shifts $B_{\text{no}}$ by 0.03 cm^3^ mol^−1^, which is barely perceptible on the scale of figure 4(b). Finally, the present work provides an internal crosscheck afforded by the two different mixtures employed. The $B_{\text{no}}$ derived from each mixture is internally consistent; the difference between the $B_{\text{no}}$ is plotted as the dotted line in figure 4(b) and has been labelled ‘consistency.’ Throughout the temperature range, the line of consistency lies within the shaded area ±0.13 cm^3^ mol^−1^, which denotes the $u\left(B_{\text{no}}\right)$ for the $x_{\text{n}}\approx 0.787$ mixture. However, the mutual uncertainty when comparing results from two mixtures is approximately two times larger than the shaded area. The larger mutual uncertainty is caused by the contribution $u\left(B_{\text{oo}}\right)$ makes to $u\left(B_{\text{no}}\right)$ derived from the $x_{\text{n}}\approx 0.200$ mixture (as discussed in the uncertainty subsection next). In any case, the consistency in $B_{\text{no}}$ derived from the two mixtures is well within the mutual uncertainty.

### 5.4. Uncertainty of the N_2_ + O_2_ results

The two results of interest from the binary mixtures are the molar refractivity $A_{\text{mix}}$ and the density cross virial coefficient $B_{\text{no}}$. These two results refer to a binary mixture whose composition has uncertainty. From equation (10), using the refractometer as a binary gas analyzer has three uncertainty components: the measured molar refractivity of the mixture, plus the reference values for the molar refractivities of nitrogen and oxygen. Measurement uncertainty for the molar refractivity adds in quadrature the apparatus baseline uncertainty $2.3\times 10^{-6}A_{\text{mix}}$ plus the $3\times 10^{-6}A_{\text{mix}}$ statistical contribution from the regression. The combined standard uncertainty is $3.8\times 10^{-6}A_{\text{mix}}$. Uncertainty in the molar refractivity of nitrogen $5.7\times 10^{-6}A_{\text{n}}$ has been established by Yang *et al* [15]. Uncertainty in the molar refractivity of oxygen $7.2\times 10^{-6}A_{\text{o}}$ was explained in section 4. Taking partial derivatives of equation (10) for the three uncertainty components and adding in quadrature gives $u\left(x_{\text{n}}\right)$ estimates of 1.5 × 10^−5^ and 1.7 × 10^−5^, for the $x_{\text{n}}\approx 0.787$ and $x_{\text{n}}\approx 0.200$ mixtures, respectively. (Uncertainty determining the mole fraction is much larger than precision because of the similarity between $A_{\text{n}}$ and $A_{\text{o}}$. This feature of an optical binary gas analyzer is analogous to the similarity of the mass problem in the more established acoustic binary gas analyzer [59].)

Uncertainty for the density virial coefficient of the mixtures $u\left(B_{\text{mix}}\right)$ adds in quadrature the statistical uncertainty of the multi-isotherm regression plus systematic conversion error $u\left(b_{\text{mix}}\right)$. The $b_{\text{mix}}\approx x_{\text{n}}b_{\text{nn}}+x_{\text{o}}b_{\text{oo}}$ has uncertainty contributions from $b_{\text{nn}}$ and $b_{\text{oo}}$, which were stated in the text above. Additionally, the arithmetic mean assumption for the cross virial coefficient $b_{\text{no}}\approx \left(b_{\text{nn}}+b_{\text{oo}}\right)/2$ is assumed to have uncertainty spanning half the range $\left|b_{\text{nn}}-b_{\text{oo}}\right|$ plus the fact that each pure component value has its respective uncertainty. As a representative value, $b_{\text{no}}=0.20(9){\text{cm}}^{3}{\;\text{mol}}^{-1}$ at 30 °C, with the number in parenthesis reflecting the uncertainty assumptions stated. Overall, the combined uncertainty is proportional to the oxygen mole fraction because $u\left(b_{\text{oo}}\right)$ is about 5 times larger than $u\left(b_{\text{nn}}\right)$. For the $x_{\text{n}}\approx 0.787$ mixture, the systematic conversion error is about two times larger than the statistical regression error; for the $x_{\text{n}}\approx 0.200$ mixture, the systematic uncertainty is three times larger than the statistical one.

From equation (12), uncertainty in the second cross virial density coefficient $B_{\text{no}}$ has contributions from the deduced $B_{\text{mix}}$, plus the pure component density virial coefficients $B_{\text{nn}}$ and $B_{\text{oo}}$, plus the mole fraction $x_{\text{n}}$. The $u\left(B_{\text{mix}}\right)$ was given in the previous paragraph, the $u\left(B_{\text{nn}}\right)$ is stated in [19], and the $u\left(B_{\text{oo}}\right)$ is indicated in table 2. Finally, $u\left(x_{\text{n}}\right)$ has been given above. Again, taking the four partial derivatives of equation (12) and adding in quadrature yields $u\left(B_{\text{no}}\right)$, which is about 0.13 cm^3^ mol^−1^ and 0.42 cm^3^ mol^−1^ for the $x_{\text{n}}\approx 0.787$ and $x_{\text{n}}\approx 0.200$ mixtures, respectively.

## 6. Natural dry air

The Ciddor formulation [9] models air as a pseudo-binary mixture of dry air and water vapor. For moist air at room conditions, the mole fraction of water vapor is about 0.01, so the dry air component contributes about 99% to the refractive index. Therefore, establishing a reference value for the refractive index of dry air is essential to the Ciddor revision.

Likewise, the density formulation of moist air [12] heavily depends on the second density virial coefficient of dry air. The present results are crucial to revising the reference formulation for the density of air, and will immediately benefit acoustic metrology performed in air [60].

### 6.1. Method for dry air

Natural dry air was supplied in a compressed cylinder. The sample was prepared at Niwot Ridge, CO USA by the National Oceanic and Atmospheric Administration. The preparation and analysis procedures supporting this sample are described by Rhoderick *et al* [61]. The mole fraction of carbon dioxide was characterized as (388.86 ± 0.15) × 10^−6^. Trace gas methane, nitrous oxide, and sulfur hexafluoride were also characterized at (1871.0 ± 0.5) × 10^−9^, (333.0 ± 0.5) × 10^−9^, (7.1 ± 0.1) × 10^−9^, respectively; the global and seasonal variability of these three trace gases is not relevant for the air refractive index formulation.

Employing a cylinder of compressed natural dry air affords tremendous utility (i.e. compared to drying room air at the point of use). Connecting the cylinder to the apparatus allowed isothermal measurements to proceed identical to the previous pure gas sections—rapid and fully automated. Measurements for the refractive index of dry air covering temperatures $\left(20<t_{90}<100\right)$ °C and pressures p<0.5MPa are unprecedented. The multi-isotherm analysis of equation (4) produced the key results for molar refractivity and the second density virial coefficient, discussed next.

### 6.2. Molar refractivity of dry air

The multi-isotherm analysis found the equation (5) coefficients to be $A_{303}=4.35698(3)\;{\text{cm}}^{3}{\;\text{mol}}^{-1}$ and $A_{\theta }=1.4(2)\times 10^{-6}$ at 0.633μm. At 1.542μm, the result was $A_{303}=4.30556(3)\;{\text{cm}}^{3}{\;\text{mol}}^{-1}$ with $A_{\theta }=1.3(2)\times 10^{-6}$. The numbers in parentheses denote statistical uncertainty only, and combined measurement uncertainty is given below.

In the context of the Ciddor revision, there are objections to using $A_{\text{R}}$ deduced by equation (4). The objection is rooted in the fact that $A_{\text{R}}$ refers to the low density limit, whereas the Ciddor formulation [9] operates on refractive index $n_{\text{as}}\to {\left[\left(1+2\rho r_{\text{m}}\right)/\left(1-\rho r_{\text{m}}\right)\right]}^{1/2}$ at some standard conditions for density. The molar refraction

(13) $$r_{\text{m}}=\frac{n^{2}-1}{n^{2}+2}\frac{ZRT}{p},$$

slightly differs from the quantity molar refractivity $A_{\text{R}}$. The measurements in this work indicate $r_{\text{m}}=4.35699(3)\;{\text{cm}}^{3}{\;\text{mol}}^{-1}$ at 0.633μm and $r_{\text{m}}=4.30558(3)\;{\text{cm}}^{3}{\;\text{mol}}^{-1}$ at 1.542μm. These reference values have been produced using the compressibility factor Z=0.9996506(2) at conditions $p=100\;\text{kPa},t_{90}=20{}^{\circ}\text{C}$, and $x_{\text{c}}=0.0389\%$. This $1.2\times 10^{-5}(n-1)$ difference between $A_{\text{R}}$ and $r_{\text{m}}$ quantities hardly matters for most applications. However, the correct estimate of $n_{\text{as}}-1$ in the Ciddor framework should use equation (13) with $r_{\text{m}}$. Adjustment to a standard mole fraction of carbon dioxide (e.g. $x_{\text{c}}=0.045\%$) should employ the mixing rule $A_{\text{mix}}=\sum_{i}A_{i}x_{i}$, and maintain the mole fraction O_2_ + CO_2_ constant [12]. The adjustment is straightforward because $A_{\text{R}}$ for carbon dioxide has been established in section 3.

### 6.3. Second density virial coefficient of dry air

**Figure 5.**
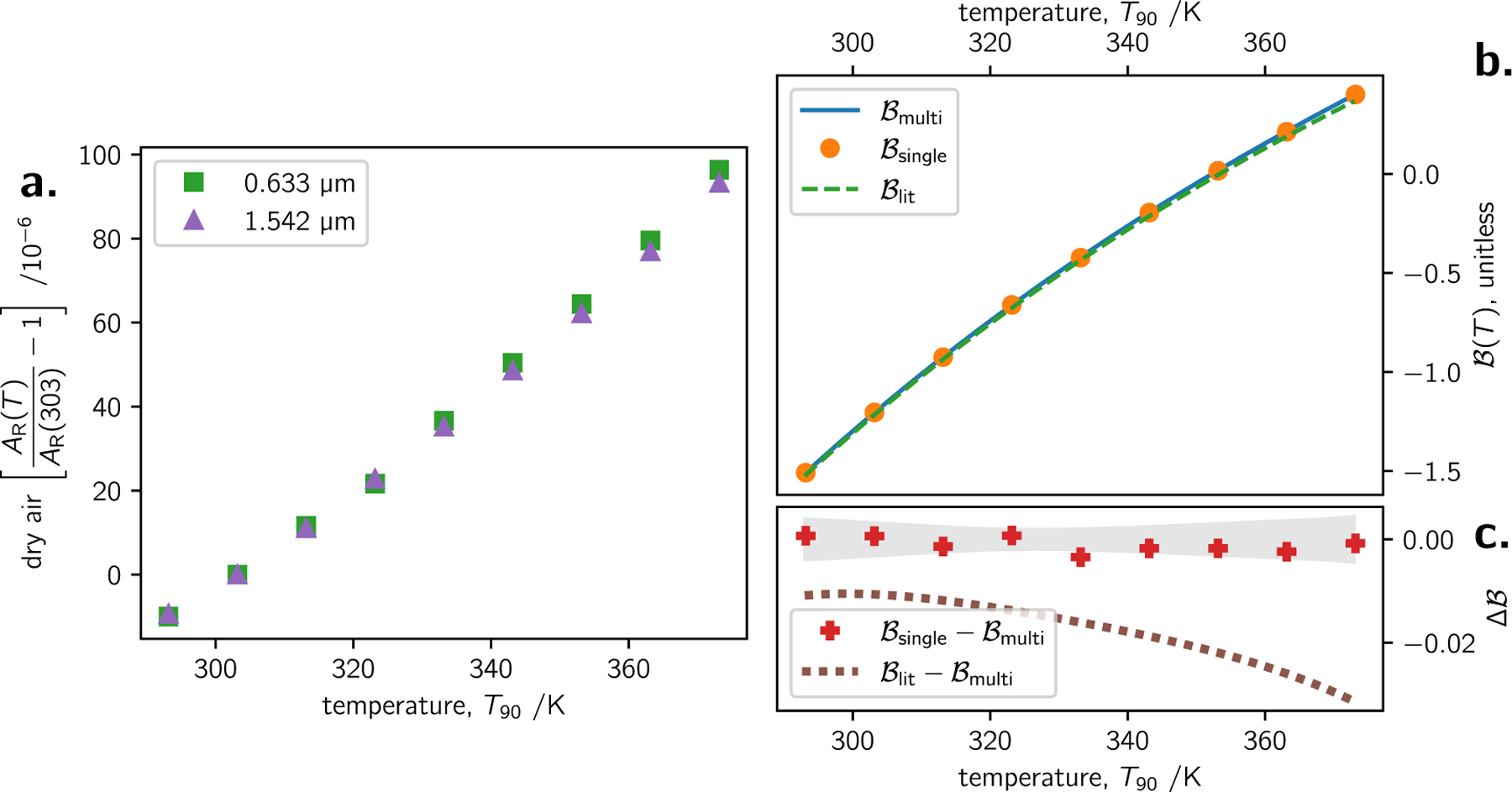
Measurement of dry air isotherms. (a) Temperature dependence of the molar refractivity. (b) The second RIGT virial coefficient ℬ. (c) Difference between this work and literature for ℬ. The shaded area denotes statistical uncertainty of this work.

The second density virial coefficient for dry air was derived from the multi-isotherm regression which identified the RIGT virial ℬ, plotted in figure 5(b). The ℬ was converted to the second density virial coefficient using equation (7) plus an estimate of the second refractivity virial coefficient $b_{\text{R}}\equiv \left(b_{\epsilon }+b_{\text{R}}^{\left(2\right)}\sigma^{2}\right)\left(1+b_{\theta }\Delta T\right)$. Dry air was approximated as a ternary mixture of N_2_ +O_2_ +Ar and $b_{\text{aa}}\approx x_{\text{n}}b_{\text{nn}}+x_{\text{o}}b_{\text{oo}}+x_{\text{r}}b_{\text{rr}}$ was used. The second refractivity virial coefficients for nitrogen $b_{\text{nn}}$ and oxygen $b_{\text{oo}}$ are stated in section 5. For argon,

$$b_{\text{rr}}/\left({\text{cm}}^{3}{\;\text{mol}}^{-1}\right)=\left(0.412+0.00321\sigma^{2}\right)\left[1-0.3\times 10^{-3}(T/\text{K}-303)\right],$$

was used, which employs $b_{\epsilon }$ and $b_{\theta }$ from the *ab initio* calculation of Garberoglio and Harvey [64], but derives $b_{\text{R}}^{\left(2\right)}$ from the method described in the appendix. The $b_{\text{rr}}$ with the updated (measured) $b_{\text{R}}^{\left(2\right)}$ is closer to the measurement of Achtermann *et al* [65] than what would be estimated by the ab initio value [64]. For the mole fractions of the ternary mixture, $x_{\text{n}}=0.78,x_{\text{o}}=0.21$, and $x_{\text{r}}=0.01$ were assumed.

The derived result for the second density virial coefficient of dry air is listed in table 4. The table compares the result against the model fit of Hyland and Wexler [62]. Their model fit from 1983 was based on older measurements, and their recommendation underlies the formulation for the density of air [66] as implemented in the Ciddor equation [9]. The agreement is excellent near ambient temperature, but the present results are more accurate, and provide key input data to revise the reference formulation for air density. Also listed in table 4 are values derived from the reference equation of state [63], in the pseudo-pure fluid version. The nominal −0.5 cm^3^ mol^−1^ offset of the equation of state is about an order of magnitude larger than the omitted influence of carbon dioxide. (The ternary mixture version of the equation of state has a nominal offset of only +0.1 cm^3^ mol^−1^.)

### 6.4. Uncertainty of the dry air results

The uncertainty of the molar refractivity has combined contribution from the baseline apparatus uncertainty plus statistical uncertainty from the isothermal regression. The reported result is for a well-characterized sample [61] of natural dry air at specified carbon dioxide content, and the trace concentrations of methane, nitrous oxide and sulfur hexafluoride are also specified; the measured refractivity has no uncertainty contribution from impurity. The statistical uncertainty on the molar refractivity is $1.6\times 10^{-6}A_{303}$. The uncertainty is primarily caused by nonlinear error in the ITS-90 temperature scale between $\left(20<t_{90}<100\right)$ °C. The combined standard uncertainty on the molar refractivity is $2.8\times 10^{-6}A_{303}$. The nonlinear error in the temperature scale also contributes the dominant systematic error $2.9\times 10^{-2}A_{\theta }$ to the temperature dependence.

Uncertainty in the second density virial coefficient combines statistical error in the multi-isotherm regression plus systematic conversion error caused by the second refractivity virial coefficient $b_{\text{R}}$. The effective contribution of statistical uncertainty is depicted by the shaded area of figure 5; for the $u\left(B_{\rho }\right)$ equivalent, recall the $3A_{\text{R}}/2$ multiplicative factor from equation (7). Depending on the temperature range, the statistical contribution is three to four times smaller than the systematic conversion error arising from the second refractivity virial coefficient. For $b_{\text{R}}$, dry air is a simple (noninteracting) mixture of N_2_ + O_2_ + Ar: a 10% uncertainty is assigned to $b_{\text{nn}}$, a 50% uncertainty is assigned to $b_{\text{oo}}$, and a 10% uncertainty is assigned to $b_{\text{rr}}$. The $u\left(b_{\text{nn}}\right)$ was stated by Achtermann *et al* [32]. The assumed uncertainty for oxygen is based on the observation that the heuristic estimate [33] of $b_{\text{oo}}$ deviates by 50% in the frequency dependence compared to what is deduced by the method described in the appendix. The $u\left(b_{\text{rr}}\right)$ is slightly larger than what was stated by Garberoglio and Harvey [64]; the slight enlargement covers observed difference in the $b_{\text{R}}^{\left(2\right)}$ coefficient inferred by this work compared to what was calculated by Garberoglio and Harvey (see the appendix). Added in quadrature with $u\left(b_{\text{nn}}\right),u\left(b_{\text{oo}}\right)$, and $u\left(b_{\text{rr}}\right)$ is the uncertainty estimate caused by the arithmetic mean assumption on the cross virial $b_{\text{no}}\approx \left(b_{\text{nn}}+b_{\text{oo}}\right)/2$. The estimated uncertainty of this assumption has been described in the context of the N_2_ + O_2_ binary mixture in section 5. Overall, the contribution of $u\left(b_{\text{oo}}\right)$ is about 30% larger than $u\left(b_{\text{nn}}\right)$; the $u\left(b_{\text{rr}}\right)$ has negligible influence. Therefore, although oxygen composes 21% of natural dry air, the large assumed $u\left(b_{\text{oo}}\right)$ is the dominant uncertainty on the derived $B_{\rho }$.

## 7. Water

Although the mole fraction of water vapor is only about 1% of room air, water plays an enlarged role in the reference formulation for refractive index. This fact has two origins: (i) historical measurements of (visible) water refractivity have disagreed by ±2%, and (ii) strong absorption in the near infrared means smooth model functions struggle to follow refractivity in detail across the visible and near infrared spectrum.

Water was already measured with the present apparatus [10, 16]. However, those measurements at the two wavelengths were separate in time. Moreover, the measurement at 0.633μm [16] had an extensive characterization of the dominant systematic error across many isotherms; by contrast, the measurement at 1.542μm [10] focused on one (high) temperature, at which the dominant systematic error was believed to have been reduced.

When revising the water portion of the Ciddor formulation, subtle issues were encountered when fitting a model to those two measurements. It was unclear if the issues were caused by measurement error or inadequacy of the model function. For this reason, measurement of ordinary water H_2_O was revisited, using a (simultaneous) two-color methodology, as now described.

### 7.1. Method for water

For water measurements, the apparatus remained much the same as Yang *et al* [15], with two minor changes. The first change is that water measurements occur below the saturation pressure, which is about 2.3 kPa at room temperature. Therefore, the piston gage of Yang *et al* was replaced by a low-pressure transducer.

With the pressure transducer installed, simultaneous measurements at 0.633μm and 1.542μm were performed. These measurements were not as extensive in pressure and temperature as previous [10, 16] because the critical information was immediately apparent from the refractivity ratio. Three isotherms were acquired at 20 °C, 60 °C, and 100 °C.

A second change to the apparatus over Yang *et al* was then pursued. The 1.542μm laser (1 kHz linewidth, external waveguide diode laser) was replaced with a widely tunable laser (100 kHz linewidth, external cavity diode laser), which allowed measurements to be performed at several wavelengths (1.52<λ<1.57)μm. (The cavity finesse remains 12% of nominal value within this wavelength range, and the reflection phase shift is well behaved.) These ‘C-band telecom’ measurements were also performed simultaneous with the 0.633μm laser remaining unchanged. Also, when measuring an isotherm, the relative frequency of the widely tunable laser was not allowed to track a resonance more than 3 × 10^−6^ distant from the vacuum resonance frequency. That is, even with the widely tunable laser, the Δf/ν of equation (1) remains effectively a change in cavity mode number when filled with gas. Consequently, group delay dispersion of the mirror coating has negligible effect across the multiple wavelengths tested.

### 7.2. Refractivity ratios of water

The chief result of interest for water is the ratio of the C-band refractivity relative to 0.633μm.

**Figure 6.**
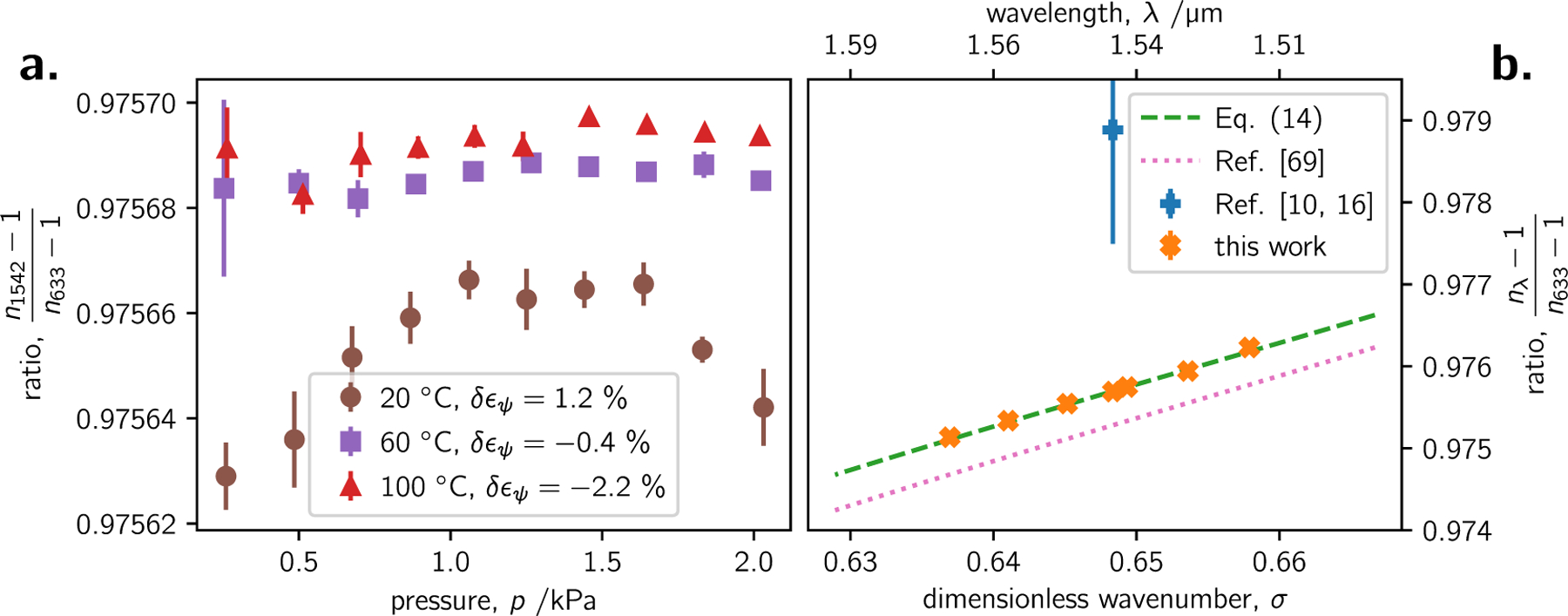
Water refractivity ratios for simultaneous two-color measurement. (a) Ratios for three different isotherms. The errorbars span standard deviation on the 10 repeat measurements at each set pressure. (b) Ratio dispersion at C-band wavelength.

For the first test, the laser systems remained identical to previous sections, and the ratio of refractivity at 1.542μm relative to 0.633μm was measured at three different isotherms. These results are presented in figure 6(a) across a range of pressures over three isotherms. The quantity plotted is

$$\frac{n_{1542}-1}{n_{633}-1}=\frac{\frac{-\Delta f_{1542}+\kappa \Delta p\nu_{1542}}{\nu_{1542}(1-\kappa \Delta p)}+\frac{2\epsilon_{\psi }}{L}\left(1+\delta \epsilon_{\psi }\right)}{\frac{-\Delta f_{633}+\kappa \Delta p\nu_{633}}{\nu_{633}(1-\kappa \Delta p)}+\frac{2\epsilon_{\psi }}{L}},$$

from equation (1) using ΔL/L≡κΔp. The approximations used over the full working equation [15] for the refractometer are unimportant. However, the variable $\delta \epsilon_{\psi }$ is important: it is a phenomenological factor that accounts for how much $\epsilon_{\psi }$ differs between wavelengths. Recall, $\epsilon_{\psi }$ was thoroughly characterized [16] at 0.633μm, but the previous 1.542μm measurement [10] used $\delta \epsilon_{\psi }=14\%$; the estimate was based on a mirror stack model that calculated how the reflection phase shift changes when voids in each dielectric layer are gradually filled by adsorbed water. In this work, $\delta \epsilon_{\psi }$ was chosen phenomenologically to ‘zero the slope’ of the refractivity ratio as a function of pressure. Although slope on the refractivity ratio also depends on dispersion in the second refractivity virial coefficient $b_{\text{R}}^{\left(2\right)}$ of water (which is unknown), at these low densities, the influence of $b_{\text{R}}^{\left(2\right)}$ should be very small. Therefore, slope on the refractivity ratio may be predominantly attributed to $\delta \epsilon_{\psi }$, and zeroing the slope gives the physically correct behavior.

High precision ratios are plotted in figure 6(a) for the three isotherms, and the phenomenological $\delta \epsilon_{\psi }$ used for each isotherm is annotated in the legend. As expected, ratio measurements become more precise as temperature increases, accordant with the underlying model for $\epsilon_{\psi }$ [16]. However, a $\delta \epsilon_{\psi }$ seemingly proportional to temperature is happenstance; the apparent temperature dependence of the ratio is also spurious. (To clarify: the refractivity ratio is temperature dependent because the temperature dependence of the molar polarizability is different at both wavelengths. However, the temperature dependency of the ratio should be much weaker than the $5.5\times 10^{-6}A_{\text{R}}/\text{K}$ for the absolute value [67].) These specious features are all related to the hysteresis of adsorption [68]. For example, if the isotherm begins in a ‘dry’ state and runs with increasing water pressure, the $\delta \epsilon_{\psi }$ takes a positive value; by contrast, beginning in a ‘wet’ state or running the isotherm with decreasing pressure gives $\delta \epsilon_{\psi }$ a negative value. Across multiple tests, $\delta \epsilon_{\psi }$ was found to take a value (2±5)%. Likewise, on the 20 °C isotherm of figure 6(a), $\delta \epsilon_{\psi }$ seems to change during the isotherm. The hysteresis of adsorption completely dominates the ratio accuracy. (Note that unpredictability in $\delta \epsilon_{\psi }$ is symptomatic of the inadequacy of the $\epsilon_{\psi }$ error model to account for hysteresis; it is not a problem with just $n_{1542}-1$, as the ratio equation above might suggest.)

Moving on to further ratio measurements: the second test used the widely tunable laser, and measured refractivity ratio at six C-band wavelengths relative to 0.633μm for the single isotherm 100 °C. The result is plotted in figure 6(b). The few 10^−5^ ratio imprecision discussed above is not observable on the ordinate scale, and the individual data points appear to perfectly follow a line. The two lines plotted in figure 6(b) are ratios produced from two model functions for the refractivity of water vapor. The dotted line offset ‘low’ is the water refractivity model due to Voronin and Zheltikov [69]. The dashed line intersecting the measured ratios is an empirically adjusted version of the Voronin and Zheltikov model. The Voronin and Zheltikov model and its adjustment is explained more in a subsection below. Note that the local dispersion dn/(dσ) is much higher than a broad spectrum Cauchy equation can follow. The result of figure 6(b) is vital to correctly implement the group index $n_{\text{g}}=n+\sigma \text{d}n/(\text{d}\sigma )$ of moist air for C-band wavelength.

Finally, the single marker offset ‘high’ in figure 6(b) is the temporally separated ratio of Egan and Yang [10, 16]. Part of that erroneous result may be attributed to the model estimate $\delta \epsilon_{\psi }=+14\%$, which was too large. The large model estimate arose from the assumption that voids in the dielectric layers are completely empty at vacuum. In reality, such an initial condition would only be approached after a high-temperature bake out. However, even acknowledging the erroneous model estimate for $\delta \epsilon_{\psi }$, the temporally separated ratio does not agree with the present work within mutual uncertainty. It is likely that the instability of the pressure transducer also contributed to the discrepant ratio. Despite the initial (thorough) characterization for the 0.633μm work, the transducer may not have held its calibration within 0.1% for the later 1.542μm work. In any case, the strength of the present work is that a refractivity ratio is largely independent of pressure and temperature. Crucially, the 0.633μm result of Egan and Yang [16] is independently supported by Schödel *et al* [70], and (to some extent) by *ab initio* calculation of the zero-frequency electric polarizability [67]. The accepted absolute value for $n_{633}-1$ makes the precision ratio especially impactful.

### 7.3. Uncertainty of the water results

The focus of the water tests was to establish the ratio of refractivity between two wavelengths, and not an absolute value of refractivity. Most of the dominant systematic uncertainties of the apparatus cancel from a ratio when the measurement is performed simultaneous at the two wavelengths.

The one systematic error that does not cancel from the ratio is captured by the variability of $\delta \epsilon_{\psi }$. As discussed in the subsection above, $\delta \epsilon_{\psi }$ was phenomenologically identified as a factor needed to make the refractivity ratio constant as a function of pressure, which is the physically correct behavior at these low densities. However, it was observed that $\delta \epsilon_{\psi }$ was somewhat unpredictable owing to the hysteresis of adsorption. The phenomenological factor was found to take a value $\left(-3<\delta \epsilon_{\psi }<7\right)\%$. At 100 °C, this level of irreproducibility gives the ratio a 1.6 × 10^−4^ range. Half the range is taken as the standard uncertainty for the systematic component of the ratio.

The statistical uncertainty was evaluated as the standard deviation of the mean for an isotherm (i.e. the sample size was ten set pressures, and each set pressure was the average of ten repeat measurements). The 100 °C isotherm gave a 5.3 × 10^−6^ statistical uncertainty for the ratio.

The combined uncertainty for the refractivity ratio at is 8.1 × 10^−5^, and is completely dominated by the systematic error caused by $\delta \epsilon_{\psi }$ irreproducibility. The mean ratio for the 100 °C isotherm is $\left(n_{1542}-1\right)/\left(n_{633}-1\right)=0.97569(8)$, with the number in parenthesis denoting the combined standard uncertainty. The mean value with its uncertainty covers all ratio data in figure 6(a). However, the apparent temperature dependence of the ratio in the present results is not believed to be real; it is a consequence of $\delta \epsilon_{\psi }$ having enlarged influence on the lower temperatures. Until knowledge of the ratio temperature dependence becomes available, it is recommended to use the mean value of the 100 °C isotherm for all temperatures $\left(0<t_{90}<200\right)$ °C. Qualitatively, one might expect the temperature dependence of the ratio to be at least an order of magnitude smaller than the $5.5\times 10^{-6}A_{\epsilon }/\text{K}$ of the static polarizability [67]. This qualitative estimate suggests that the ratio would only change value by 11 × 10^−5^ across $\left(0<t_{90}<200\right)$ °C, which is only slightly larger than the uncertainty of the present recommendation. A semiquantitative estimate notes that the change in ratio between all data on the 60 °C and 100 °C isotherms is only 1.6 ×10^−7^ K^−1^.

### 7.4. Empirically adjusted dispersion of water

Voronin and Zheltikov [69] developed a sophisticated model for the dispersion of water vapor. The model followed a generalized Sellmeier equation, in which absorption line lists from HITRAN were transformed into (infrared) refractive index and combined with semiempirical descriptions of (visible) refractive index from the dipole oscillator strength distributions. Their formulation has the form

(14) $$A_{\text{R}}(\sigma )=Q\sum_{i=1}^{8}\left(\frac{S_{\text{a}i}}{\Omega_{\text{a}i}^{2}-\sigma^{2}}+\frac{S_{\text{b}i}}{\Omega_{\text{b}i}^{2}-\sigma^{2}}\right).$$

It is well-established [16, 70] that the Voronin and Zheltikov model for water vapor refractivity is too low by 2.2% of absolute value at 0.633μm. This fact suggests problems with the high frequency behavior of the Sellmeier (i.e. the strengths of the ultraviolet oscillator-pair derived from the dipole oscillator strength distributions). Moreover, the low value for the C-band ratio evident figure 6(b) may also be attributed to the ultraviolet oscillator-pair (i.e. the strengths *and* locations of the ultraviolet oscillatorpair).

The strengths and locations of the ultraviolet oscillator-pair of Voronin and Zheltikov were empirically adjusted to satisfy two conditions: (i) the absolute value of $A_{\text{R}}(\sigma =1.58)$ must intersect the reference value of Egan and Yang [16], and (ii) the ratio $A_{\text{R}}(\sigma =0.65)/A_{\text{R}}(\sigma =1.58)$ must intersect this work. It was found that both conditions were satisfied by increasing the oscillator-pair strengths by 3.61% and shifting up the oscillator-pair frequency by 0.72%. Updated coefficients for equation (14) are listed in table 5. The only adjustments over Voronin and Zheltikov are to the ultraviolet oscillator-pair in row i=8.

## 8. Conclusion

A suite of refractivity measurements has been reported, whose purpose is to enable revision of Ciddor’s formulation [9] for the refractive index of air. The measurements have been performed at two wavelengths—0.633μm and 1.542μm—which will tightly constrain dispersion for the revised formulation.

Compressed gases studied included carbon dioxide, oxygen, binary mixtures of nitrogen–oxygen, and natural dry air. In addition to the molar refractivities of these gases, the work also derived the second density virial coefficient. In some cases (e.g. oxygen), this work contributes information on gas properties which has hitherto been scarce.

The water vapor measurements focused on the ratio of refractivity between the two wavelengths. The ratio optimized the Voronin and Zheltikov [69] model, which is potentially valid for wavelengths out to 14μm. The optimized model for water vapor refractivity is essential for an accurate formulation of the group refractive index at telecom wavelength.

## Figures and Tables

**Figure 7.**
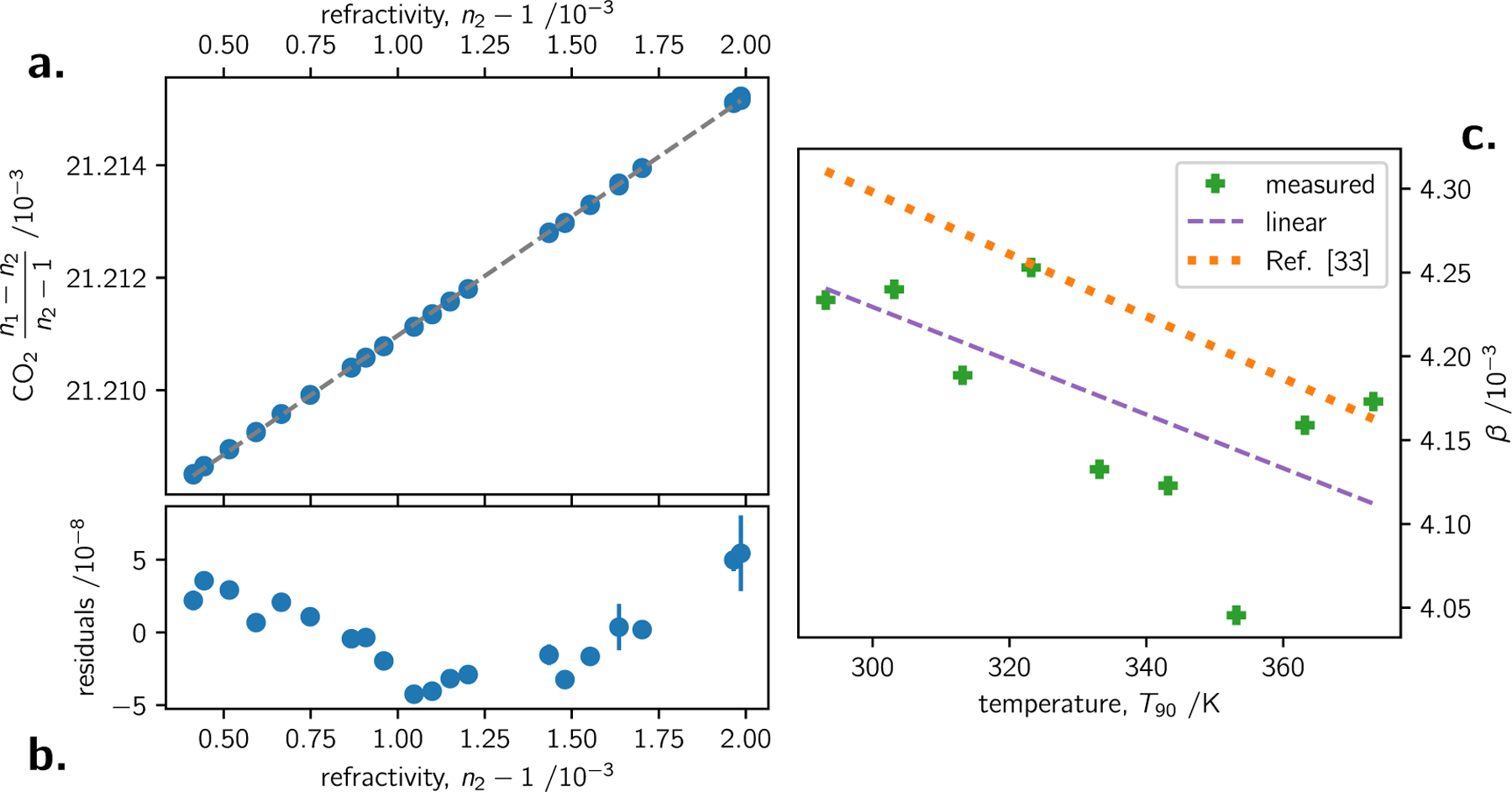
Analysis of the refractivity ratio for carbon dioxide. (a) Deviation of the ratio from unity on the 30 °C isotherm, and (b) residuals from the fit of equation (15). (c) Fit parameter β as a function of temperature.

**Table 1.** Second density virial coefficient of carbon dioxide derived from this work. Also listed are values for the adjusted *ab initio* calculation of Hellmann [25] and the reference equation of state by Span and Wagner [31]. Numbers in parentheses denote standard uncertainty.

	$B_{\rho }/\left({\text{cm}}^{3}{\;\text{mol}}^{-1}\right)$		
$T_{90}/K$	this work	Ref. [25]	Ref. [31]
293.15	−127.65(5)	−128.0(9)	−127.9
303.15	−118.21(4)	−118.4(8)	−118.4
313.15	−109.64(3)	−109.8(8)	−109.8
323.15	−101.86(3)	−102.0(8)	−102.1
333.15	−94.78(4)	−94.9(7)	−95.0
343.15	−88.32(4)	−88.4(8)	−88.5
353.15	−82.40(4)	−82.4(6)	−82.6
363.15	−76.93(4)	−76.9(8)	−77.1
373.15	−71.83(5)	−71.8(6)	−72.1

**Table 2.** Second density virial coefficient of oxygen derived from this work. Also listed are values from the *ab initio* calculation of Hellmann [37] and the reference equation of state by Stewart *et al* [42]. Numbers in parentheses denote standard uncertainty.

	$B_{\rho }/\left({\text{cm}}^{3}{\;\text{mol}}^{-1}\right)$		
$T_{90}/K$	this work	Ref. [37]	Ref. [42]
293.15	−17.0(1)	−17.2(5)	−17.4
303.15	−14.9(1)	−15.1(5)	−15.3
313.15	−12.9(1)	−13.1(5)	−13.3
323.15	−11.1(1)	−11.3(5)	−11.5
333.15	−9.4(1)	−9.6(5)	−9.8
343.15	−7.8(1)	−8.0(5)	−8.2
353.15	−6.4(1)	−6.5(5)	−6.7
363.15	−5.0(1)	−5.2(5)	−5.3
373.15	−3.7(1)	−3.9(5)	−4.0

**Table 3.** Second density virial and cross virial coefficient for binary mixtures of N_2_ + O_2_. The column heading specifies the nitrogen mole fraction $x_{\text{n}}$ deduced by equation (10). Numbers in parentheses denote standard uncertainty.

	$B_{\text{mix}}/\left({\text{cm}}^{3}{\;\text{mol}}^{-1}\right)$		
$T_{90}/K$	$x_{\text{n}}=0.78706$	$x_{\text{n}}=0.19971$	$B_{\text{no}}/\left({\text{cm}}^{3}{\;\text{mol}}^{-1}\right)$
293.15	−8.48(4)	−14.96(8)	−12.2(2)
303.15	−6.50(4)	−12.90(8)	−10.1(1)
313.15	−4.65(4)	−10.98(8)	−8.2(1)
323.15	−2.95(3)	−9.18(8)	−6.4(1)
333.15	−1.36(3)	−7.51(8)	−4.7(1)
343.15	0.12(3)	−5.95(8)	−3.2(1)
353.15	1.50(4)	−4.50(8)	−1.8(1)
363.15	2.79(4)	−3.15(8)	−0.4(1)
373.15	4.01(4)	−1.90(9)	0.9(1)

**Table 4.** Second density virial coefficient of natural dry air from this work compared to the data fit function of Hyland and Wexler [62] and the reference equation of state by Lemmon *et al* [63]. Numbers in parentheses denote standard uncertainty.

	$B_{\rho }/\left({\text{cm}}^{3}{\;\text{mol}}^{-1}\right)$		
$T_{90}/K$	this work	Ref. [62]	Ref. [63]
293.15	−8.57(4)	−8.64(25)	−9.1
303.15	−6.58(4)	−6.65(25)	−7.2
313.15	−4.74(3)	−4.82(25)	−5.3
323.15	−3.03(3)	−3.12(26)	−3.7
333.15	−1.44(3)	−1.55(26)	−2.1
343.15	0.03(3)	−0.09(26)	−0.6
353.15	1.41(4)	1.27(26)	0.7
363.15	2.71(4)	2.54(26)	2.0
373.15	3.93(4)	3.72(26)	3.2

**Table 5.** Coefficients for the water vapor Sellmeier equation of equation (14) at 20 °C. All coefficients are dimensionless. The scale factor $Q=360.115{\;\text{cm}}^{3}{\;\text{mol}}^{-1}$ gives dimensions of $A_{\text{R}}$ in SI units.

i	$S_{ai}$	${𝛀}_{ai}$	$S_{bi}$	${𝛀}_{bi}$
1	2.945 × 10^−5^	0.02089	6.583 × 10^−8^	0.06023
2	3.273 × 10^−6^	0.14883	3.094 × 10^−6^	0.17452
3	1.862 × 10^−6^	0.36028	2.788 × 10^−6^	0.38484
4	2.544 × 10^−7^	0.54478	2.181 × 10^−7^	0.52499
5	1.126 × 10^−7^	0.70542	2.336 × 10^−7^	0.73276
6	6.856 × 10^−9^	0.87313	9.479 × 10^−9^	0.89031
7	1.985 × 10^−9^	1.05515	2.882 × 10^−9^	1.06942
8	0.26718980	7.86916	4.913464	28.84126

## Data Availability

The data that support the findings of this study are openly available at the following URL/DOI: https://doi.org/10.18434/mds2-4155 [35].

## Appendix. Constraining $b_{\text{R}}(\sigma ,T)$

Following ideas from Keeling [71], when refractivity is measured along an isotherm at two frequencies $\sigma_{1}>\sigma_{2}$, the ratio may be fit

(15)
$$\frac{n_{1}-1}{n_{2}-1}=\alpha +\beta \left(n_{2}-1\right)+\cdots .$$

Throughout this appendix the subscripts ‘1’ and ‘2’ denote a quantity at frequency $\sigma_{1}$ or $\sigma_{2}$, respectively. The ρ in equation (2) may be expressed with a series approximation in the independent variable

(16)
$$\rho =\frac{2}{3A_{2}}\left(n_{2}-1\right)-\frac{A_{2}+4b_{2}}{9A_{2}^{2}}{\left(n_{2}-1\right)}^{2}+\cdots ,$$

Reevaluating equation (2) with the Lorentz–Lorenz quotient series expanded in $n_{1}-1$ and the density series expanded in $n_{2}-1$, the coefficients of equation (15) are

(17)
$$\alpha =\frac{A_{1}}{A_{2}}\;\beta =\frac{A_{1}}{6A_{2}^{2}}\left(A_{1}-A_{2}+4\Delta b\right),$$

with $\Delta b=b_{1}-b_{2}$.

The preceding remarks are powerful in two ways. The first is that an analysis in the independent variable $n_{2}-1$ avoids the imprecision of density (i.e. the measured pressure and temperature of the gas). The equation (15) analysis applied to the carbon dioxide isotherm at 30 °C is plotted in Fig 7 (a). The standard deviation on the fractional residuals from a linear fit are 2.8 × 10^−8^, almost a factor of 100 smaller than density imprecision! The second strength of the β-analysis is to constrain both the frequency and temperature dependencies of $b_{\text{R}}(\sigma ,T)$. With an approximation error less than 10%, β allows an inference of the frequency dependence of the second refractivity virial coefficient via

(18)
$$b_{\text{R}}^{\left(2\right)}\approx \frac{6A_{2}\beta -A_{1}^{2}+A_{1}A_{2}}{4A_{1}\left(\sigma_{1}^{2}-\sigma_{2}^{2}\right)}.$$

**Table 6.** Parameter β for different gas species between wavelengths 0.633μm and 1.542μm at 30 °C. The ultraviolet results of Keeling [71] have been reduced by (3.94^2^ − 2.29^2^)*/*(1.58^2^ − 0.65^2^)≈ 4.95 to reflect a first-order dependence in $\sigma^{2}$. The numbers in parentheses denote standard uncertainty.

	$\beta /10^{-3}$				
species	this apparatus		ultraviolet [71]	heuristic [33]	*ab initio* [64]
He	−1.20(25)	[15]		−0.18	−1.20
Ne	−0.70(25)	[15]		0.20	−0.67
Ar	2.62(25)	[15]	2.99	2.81	1.93
${\text{N}}_{2}$	2.17(25)	[15]	2.71	2.25	
${\text{O}}_{2}$	4.73(25)			3.21	
${\text{CO}}_{2}$	4.83(25)		4.21	4.29	
dry air	3.09(25)		3.17		

The β(T) result for the nine carbon dioxide isotherms is plotted in Fig 7(c). The $b_{\text{R}}^{\left(2\right)}(T)$ may be inferred by adjusting the Δb in equation (17) so that β ‘fits’ the data. The magnitude of $b_{\text{R}}^{\left(2\right)}$ offsets β relative to the measured data; the temperature dependence of $b_{\text{R}}^{\left(2\right)}$ changes the slope of β relative to the data. So, one ratio of measured refractivities tightly constrains $b_{\text{R}}(\sigma ,T)$. Also plotted in figure 7 (c) is the heuristic estimate of Hohm [33] for carbon dioxide. Evidently, the heuristic estimate identifies the temperature dependence, but the $b_{\text{R}}^{\left(2\right)}$ of Hohm is about 2% larger than the present work.

A β-analysis for a variety of gases measured by the present apparatus may be compared with different literature sources, and is given in table 6. All tabulated values have been estimated for the operating wavelengths 0.633μm and 1.542μm of the present apparatus. The preference to report β rather than $b_{\text{R}}^{\left(2\right)}$ is because β is the measured quantity; equation (18) allows conversion between the two. For the noble gases, agreement is excellent between the present apparatus and *ab initio* calculation of helium; agreement becomes progressively worse for the larger atoms. On the one hand, large disagreement in argon is expected given limitation in the *ab initio* knowledge of the interaction polarizability and its dispersion. On the other hand, uncertainty in the experimental determination of β is about 10% on the argon value; experiment and calculation do not overlap within the expanded uncertainty of the measurement. [Measurement uncertainty is dominated by statistics; the contribution of the second order terms in Δb are five times smaller than regression error.] For argon, the ‘high’ β result from the present apparatus is supported by an independent measurement performed at ultraviolet wavelengths due to Keeling [71]. The β from Keeling are expected to be slightly higher than this work because the (unknown) $\sigma^{4}$ proportionality would contribute at ultraviolet wavelength; the inferred values in table 6 assume $\sigma^{2}$ proportionality. To be clear, equation (18) means differences among β in table 6 reflect differences in $b_{\text{R}}^{\left(2\right)}$, and say nothing about $b_{\epsilon }$. A determination of $b_{\epsilon }$ might be possible with a third wavelength.
